# Development of a central nervous system axonal myelination assay for high throughput screening

**DOI:** 10.1186/s12868-016-0250-2

**Published:** 2016-04-22

**Authors:** Karen D. Lariosa-Willingham, Elen S. Rosler, Jay S. Tung, Jason C. Dugas, Tassie L. Collins, Dmitri Leonoudakis

**Affiliations:** Translational Medicine Center, Myelin Repair Foundation, Sunnyvale, CA 94085 USA; Teva Pharmaceuticals, Biologics and CNS Discovery, Redwood City, CA 94063 USA; Alios BioPharma, South San Francisco, CA 94080 USA; Rigel Pharmaceuticals, South San Francisco, CA 94080 USA; NGM Biopharmaceuticals, Inc., South San Francisco, CA 94080 USA

**Keywords:** Myelination, Oligodendrocyte, High throughput, Drug screening, Differentiation, Primary cell-based assay, Image analysis, Multiple sclerosis, Myelin basic protein

## Abstract

**Background:**

Regeneration of new myelin is impaired in persistent multiple sclerosis (MS) lesions, leaving neurons unable to function properly and subject to further degeneration. Current MS therapies attempt to ameliorate autoimmune-mediated demyelination, but none directly promote the regeneration of lost and damaged myelin of the central nervous system (CNS). Development of new drugs that stimulate remyelination has been hampered by the inability to evaluate axonal myelination in a rapid CNS culture system.

**Results:**

We established a high throughput cell-based assay to identify compounds that promote myelination. Culture methods were developed for initiating myelination in vitro using primary embryonic rat cortical cells. We developed an immunofluorescent phenotypic image analysis method to quantify the morphological alignment of myelin characteristic of the initiation of myelination. Using γ-secretase inhibitors as promoters of myelination, the optimal growth, time course and compound treatment conditions were established in a 96 well plate format. We have characterized the cortical myelination assay by evaluating the cellular composition of the cultures and expression of markers of differentiation over the time course of the assay. We have validated the assay scalability and consistency by screening the NIH clinical collection library of 727 compounds and identified ten compounds that promote myelination. Half maximal effective concentration (EC_50_) values for these compounds were determined to rank them according to potency.

**Conclusions:**

We have designed the first high capacity in vitro assay that assesses myelination of live axons. This assay will be ideal for screening large compound libraries to identify new drugs that stimulate myelination. Identification of agents capable of promoting the myelination of axons will likely lead to the development of new therapeutics for MS patients.

**Electronic supplementary material:**

The online version of this article (doi:10.1186/s12868-016-0250-2) contains supplementary material, which is available to authorized users.

## Background

Multiple sclerosis (MS) is the most common disabling neurological disease of young adults; once established, it persists for the remainder of a person’s life [1]. The initial triggering events which lead to MS remain unknown and there is no cure. In MS, central nervous system (CNS) lesions form as a result of immune-mediated destruction of myelin sheaths that insulate and protect axons, resulting in loss of function and, ultimately, progressive neurodegeneration and permanent neurological decline. Current MS therapeutics mainly target the autoimmune response that damages myelin sheaths. Although effective in reducing relapses in early disease, none of them prevent long-term disease progression and none are effective in treating progressive forms of MS. A major unmet medical need in MS is the availability of therapeutics that directly protect myelin or promote new myelin formation to maintain nerve function, prevent neurodegeneration, and restore lost function in MS patients.

A key deficit in the development of myelin repair therapeutics is the lack of a high throughput axonal myelination assay suitable for drug discovery. The closest available high throughput systems assess differentiation of purified oligodendrocyte precursor cells (OPC) or oligodendrocyte (OL) wrapping of inert, non-biological substrates, neither of which incorporate the complex cellular interactions between OPC/OLs and live axons [2–4]. Existing myelination assays are very low throughput, time consuming, and are designed for exploring basic research themes rather than to drive drug development decisions. Development of an assay that evaluates axonal myelination, incorporates the complete repertoire of CNS cells, and can be performed at a scale and reproducibility that permits testing large numbers of potential drugs would provide a major advance in the efforts to develop remyelination-promoting therapeutics for MS patients. We describe here the development of such an assay and utilize it to identify potential myelin repair therapeutics.

In an effort to create a myelination assay more amenable to higher throughput compound screening, we used embryonic rat cortex to develop, optimize, and validate an in vitro myelination assay [5, 6] which can be utilized for chemical library screening. The culture system was miniaturized into a 96-well plate format enabling high throughput liquid handling, automated image acquisition and analysis of myelinating co-cultures. It has previously been shown that inhibition of the γ-secretase protease activity promotes differentiation of OPCs and myelination of retinal ganglion cells (RGC) in RGC-OPC co-cultures [7–9]. Based on this published work, we used the γ-secretase inhibitor (GSI), *N*-[*N*-(3,5-difluorophenacetyl)-l-alanyl]-*S*-phenylglycine t-butyl ester (DAPT) as a positive control in our cortical co-cultures [9], and confirmed that the assay allows for the quantification of early axonal myelination in a dose-dependent manner. This assay identifies compounds which are not active in a pure primary OPC differentiation assay [3, 10] but are capable of promoting re-myelination in vivo [11]. We have used this myelination assay to screen the NIH clinical collection library of small molecules. Because this mixed CNS cell assay platform more closely mimics the complex cellular interactions in the CNS in vivo, it will also likely be scalable and adaptable to the high throughput screening of other neurological disease therapeutics in future studies.

## Results

### Development of the embryonic cortical cell co-culture assay

In an effort to move a low-throughput, well-established myelination cell culture technique to a format suitable for higher throughput screening applications, we miniaturized and automated a previously described RGC-OPC co-culture technique [9]. Unfortunately, the low yield of RGCs and lack of assay robustness from each preparation makes this co-culture myelination assay unsuited for high-throughput compound screening (Additional file 1: Fig. S1A). We therefore sought another source of tissue where numbers of neurons would not be limiting.

For increased yield of primary neurons, we chose the cortex of embryonic day 18 (E18) rats as an abundant source of relatively homogeneous brain cells with well-established culture methods [5, 6] (see “Methods”). From one litter, enough cells can easily be generated for high throughput drug screening applications (~30 × 10^6^ cells/cortex; Additional file 1: Fig. S1B).

**Fig. 1:**
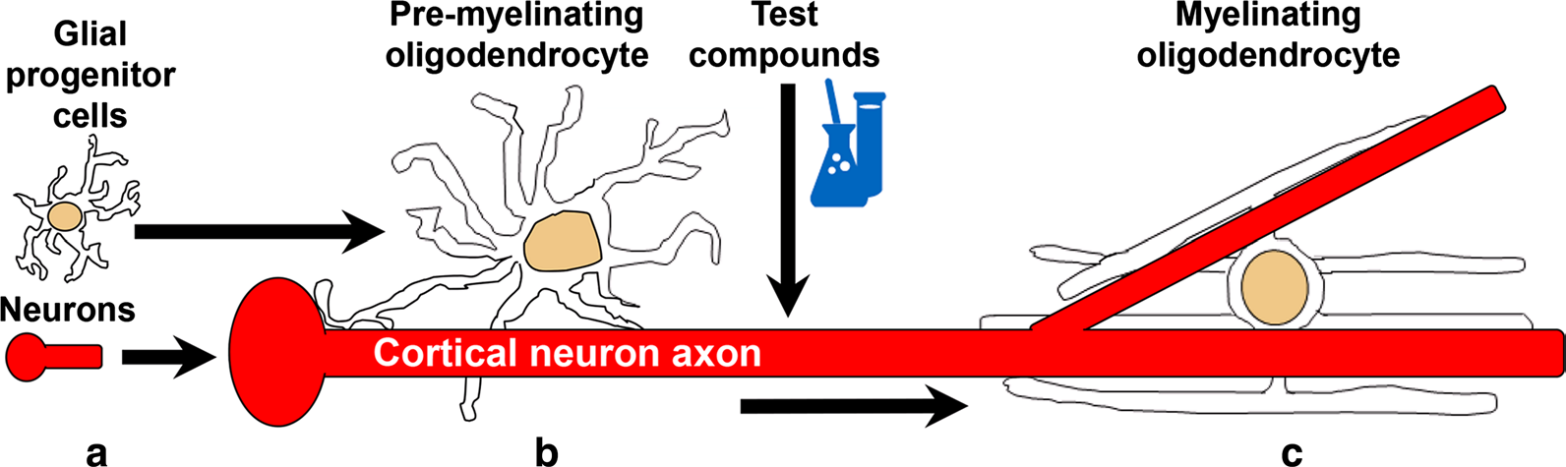
Flow scheme for the cortical cell myelination assay. **a** Dissociated cells from the cortex containing neurons and glial progenitor cells were cultured from E18 rat embryos onto poly-d-lysine/laminin coated 96-well plates. **b** On DIV4, when axonal projections (*red*) are apparent in the neuronal population, the growing co-culture was changed to MyM media to induce OL differentiation and initiate myelination. The following day test compounds were added and cultures were left undisturbed for an additional 8 days. **c** Cells were fixed and immunostained for MBP, Olig2 and DAPI on DIV13. Images were acquired using automated microscopy and scored phenotypically for myelination as described in the “Methods”

**Fig. 2:**
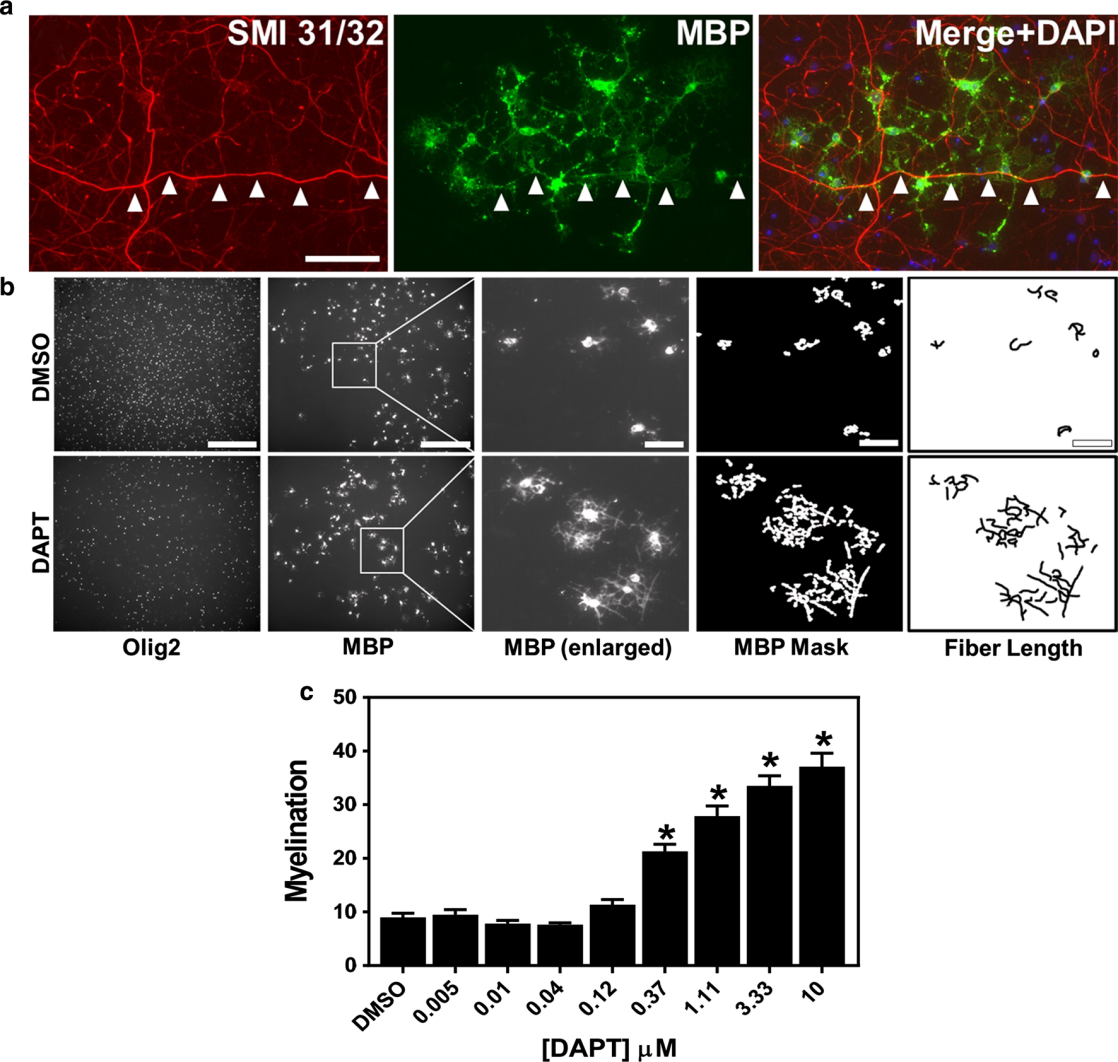
Oligodendrocyte processes align with cortical axons and γ-secretase inhibitors (GSIs) facilitate myelination. Cortical co-cultures were treated with the GSI, DAPT or DMSO as described in Fig. 1. **a** On 13 DIV, cells were fixed and stained with antibodies to the axon marker SMI 31/32 neurofilament protein (*red*) and MBP (*green*). Image at the right is a composite of the SMI 31/32, MBP, and DAPI. *Arrowheads* indicate regions of MBP alignment with axon. *Bar* 100 µm. **b** *Left two panels* show entire image fields taken from a 96-well plate immunostained for Olig2 and MBP. *Bars* 200 µm. *Boxed regions* are enlarged in the *middle panel* to show morphological detail of MBP-stained OLs. *Bar* 50 µm. *Two images at right* depict the digital mask of MBP staining intensity of the adjacent image (*middle panel*) and the *far right image* are tracings of MBP alignment used to calculate fiber length. *Bars* 50 µm. **c** Raw data from three DAPT dose response experiments was quantified from images as in **b** and compiled from n = 3 experiments, 80 image fields per concentration, mean ± SEM. *Asterisk* (*) denotes P values versus DMSO of <0.0001; ANOVA with Bonferroni post hoc test. There was a significant effect of four compound concentrations compared to DMSO [F(9, 19) = 83.82, P < 0.0001]. Post hoc comparisons indicated that the mean score for the concentrations 0.37 μM (M = 21.32, SEM = 1.3), 1.11 μM (M = 27.88, SEM = 1.9), 3.33 μM (M = 33.51, SEM = 1.9), 10 μM (M = 37.1, SEM = 2.5) was significantly different than DMSO

Following the differentiation and growth of neurons and glia for 5 days, we determined that the differentiation and early myelination of exogenously added OPCs proceeded optimally when the growth medium was switched from NB/N21 to an OPC-supporting myelination medium (MyM). With this growth medium, we observed that it was not necessary to add exogenous OPCs as we could readily identify mature and axon ensheathing OLs that had differentiated from the embryonic cortical preparation, presumably from neural precursor cells and/or OPCs. We actually found that the differentiating OPCs already present in the cultures produced better myelination (Additional file 2: Fig. S2, see below and methods for quantification of myelination). Finally, we determined that the optimal time course for myelination to proceed was 8 days after test compound addition and 13 DIV total (Additional file 3: Fig. S3). Figure 1 depicts the flow scheme of the embryonic cortical cell assay. At this early myelination time point, we observed MBP staining aligning with SMI 31/32 axon staining, indicating that indeed OLs are contacting and aligning with axons (Fig. 2a).

**Fig. 3:**
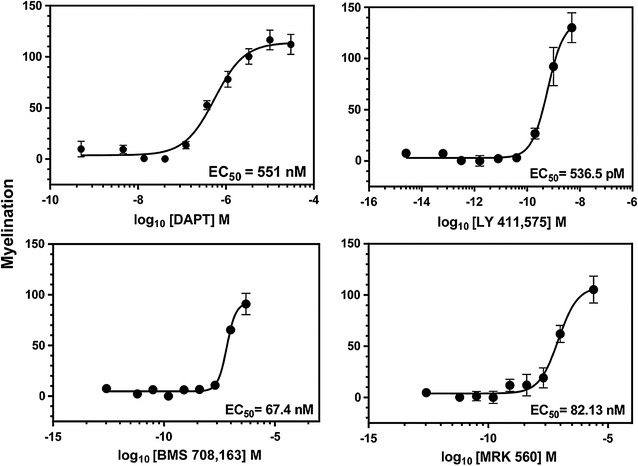
Half maximal effective concentration determination of four different GSIs for the promotion of myelination in the cortical culture assay. Dose response data confirm the activity of GSIs and enable the calculation of the EC_50_ value for each compound. Cortical cultures were treated for 8 days with DAPT, LY 411,575, BMS 708,163 or MRK 560 and immunostained for MBP, Olig2 and DAPI. Dose–response curve for DAPT is compiled from n = 3 experiments, 80 image fields per concentration. Representative dose–response curves for LY 411,575, BMS 708,163 and MRK 560 are 32 image fields per concentration, mean ± SEM. Respective EC_50_ values are shown in the legend

We utilized the γ-secretase inhibitor, DAPT, a known enhancer of myelination [9, 11] as a positive control to test our assay system and establish an automated morphology analysis. After compound treatment, cells were stained for the OL lineage marker, Olig2, myelin basic protein (MBP) to stain mature OLs, and the nuclear dye, DAPI, and imaged. Myelination was scored by quantifying the characteristic change of morphology of OLs when ensheathing axons—from many branched, flattened, and diffusely MBP stained processes to condensed and aligned MBP-positive fibers. For each high resolution 10× image, we quantified the total length of contiguous, aligned MBP staining (fiber length)/number of Olig2-positive (Olig2^+^) nuclei, referred to as myelination). Figure 2b demonstrates the digital mask created by our protocol used in the fiber length calculation. With these methods, we determined significant dose-dependent increases in myelination with DAPT (Fig. 2c). Importantly, we were able to determine reproducible EC_50_ values of four GSI compounds, DAPT, LY411,575, BMS 708,163, and MRK560, allowing the ranking of compounds (Fig. 3a–d, Table 1). GSI-mediated facilitation of myelination was only observed in the presence of live axons and had no effect on the differentiation of purified OPCs grown in isolation (Additional file 4: Fig. S4). We tested two other compounds identified from published high throughput library screens that promote OL differentiation in cultures containing purified OPCs, benztropine and clemastine [2, 3]. As expected, these compounds demonstrated significant OL differentiation in our acutely prepared OL differentiation assay (Additional file 4: Fig. S4). However, in our cortical myelination assay, benztropine and clemastine did not promote myelination (Additional file 5: Fig. S5). This data demonstrates that the cortical myelination assay identifies novel compounds with myelination activity distinct from compounds that solely promote OL differentiation.

**Table 1:** Confirmed hits from the NCC library screen

Known mechanism of action	Compound	EC_50_ μM_avg_^b^
Kinase inhibitor	Imatinib mesylate	1.4
Anti-cholinergic	Atracurium Besylate	5.3
Mitotic inhibitors	Docetaxel	0.1
	Methotrexate	0.1
	Tegafur	2.7
	Artesunate	3.3
Ion channels	Zu-capsaicin	4.7
	Amiloride	8.9
	Oxcarbazepine	14.7
Na^2+^/K^+^ ATPase inhibitor	Digoxin	11.3
γ-secretase inhibitors	LY 411, 575^a^	0.00053
	BMS 708, 163^a^	0.067
	MRK 560^a^	0.082
	DAPT^a^	0.55

^a^Compounds not part of the NCC library screen

^b^N = ≥ 2

### Long-term characterization of cortical myelination cultures

**Fig. 4:**
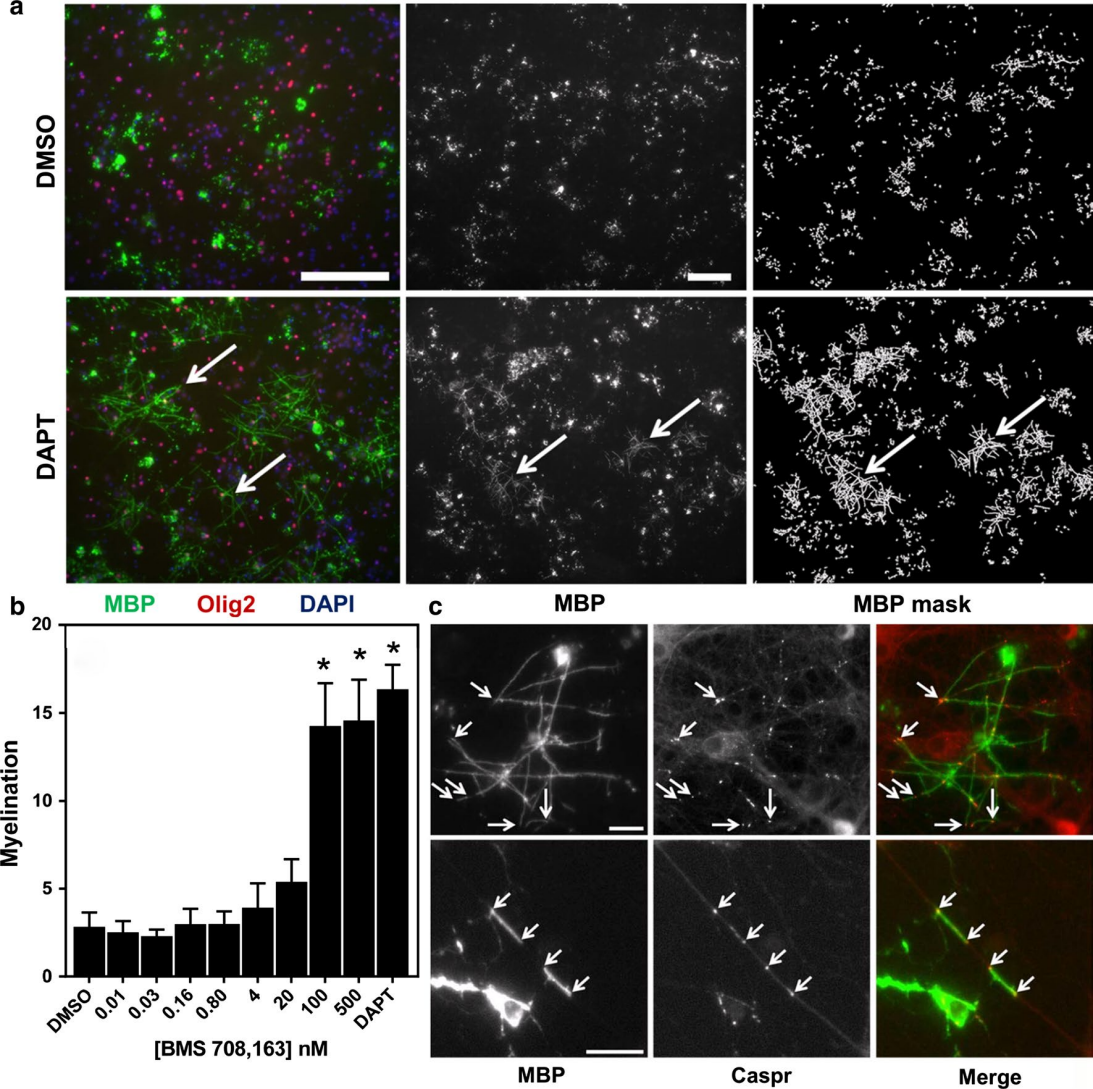
Long term cortical cultures demonstrate persistent GSI-induced enhancement of myelination and initiation of axonal node of Ranvier formation. On DIV5, cortical cultures were treated with DAPT or DMSO for 8 days, media was changed weekly thereafter without compound, and cells fixed on DIV28. **a** *Left panels* show triple immunostaining of MBP (*green*), Olig2 (*red*), and DAPI (*blue*). *Red* overlaid with *blue* appears *pink*. *Right panels* show digital masks created from MBP-stained images in the *center panel*. Masks were used for quantification of fiber length. *Bars* 100 µm. *Arrows* indicate areas with significant myelination. **b** Quantification of myelination showing raw data in 28 DIV cortical cultures as in **a**. Representative data shown is averaged from 16 image fields per concentration, mean ± SEM. *Asterisk* (*) denotes P values versus DMSO of < 0.0001; ANOVA with Dunnett’s post hoc test. There was a significant effect of three compound concentrations compared to DMSO [F(9, 27) = 17.50, P < 0.0001]. Post hoc comparisons indicated that the mean score for the concentrations 100 nM (M = 14.26, 2.433), 500 nM (M = 14.57, 2.32), and DAPT (M = 16.35, 1.39) was significantly different than DMSO. **c** Cortical co-cultures were grown for a total of 21 days, fixed, and immunostained for MBP (*green*, merged image) and the paranode-localized protein Caspr (*red*, merged image). Note the accumulation of Caspr protein at the edges of myelinated axon segments (*arrows*). *Bar*, *upper panels* 100 μm. *Bar*, *lower panels* 50 μm

To test whether the enhanced early myelination effects of GSIs had longer lasting effects with the single 8 day drug treatment course, cortical cultures were treated as described followed by 2 weeks of half medium changes with fresh MyM without GSI compound. These GSI-treated cultures demonstrated robust MBP alignment compared to DMSO vehicle controls (Fig. 4a, b). Additionally, we tested these cortical cultures for their ability to initiate the formation of axonal nodes of Ranvier, essential to action potential propagation in functionally myelinated axons. As an indicator of early node formation, we immunostained these longer term cultures with antibodies to the paranode-localized protein, Caspr [12] along with anti-MBP antibodies. Figure 4c demonstrates the accumulation of Caspr protein at the edges of myelinated axon segments indicating that the initiation of node formation was induced by contact with OL myelin. These data demonstrate the ability of cortical cultures to form robustly myelinated axon segments and initiate node formation which is enhanced by an early, single dose treatment with GSIs.

### Cellular composition of cortical myelination cultures

To determine the composition of these cortical cultures, we used well-established cell type marker antibodies to identify different cell populations. Cultures were grown using the culture conditions described above, and fixed on DIV13. All cultures were stained with the nuclear marker DAPI to identify the total population of all the cells in culture. To identify the neuronal population, we used anti-NeuN antibodies to identify neuronal nuclei, as well as anti-MAP2 and anti-SMI 31/32 neurofilament antibodies to assess the health and extent of dendrite and axon formation, respectively. Imaging of neurons in these cultures demonstrated mature cortical neurons with well-developed dendrites and a dense bed of axons (Additional file 6: Fig. S6). In addition to NeuN for the identification of neurons, we also used anti-Olig2 antibodies to identify OPC/OLs and anti-GFAP antibodies to identify astrocytes. We then quantified the percentage of each of these cell types in this cortical co-culture preparation as a percentage of the total cell population identified with DAPI nuclear staining of all cells. We found that the cell composition under these culture conditions was 23 % neurons, 46 % astrocytes, 22 % OPCs/OLs, and 9 % unidentified cells.

In order to better understand how OLs differentiate and develop in these cultures, we stained and imaged DIV5 cultures to assess the cell composition of our cultures on the day of test compound addition. At this stage, the cultures contained ~50 % neurons, having already generated an axon network (Additional file 7: Fig. S7). Since our cultures were derived from embryonic cortex, we used the bi-potent O2A glial progenitor antibody marker A2B5 [13, 14], to identify glial progenitors still capable of differentiating. DIV5 cultures contained abundant A2B5 positive cells which were not observed at DIV13 (Additional file 8: Fig. S8). There were relatively few astrocytes (positively staining for GFAP) and differentiated OLs (positively staining for MBP, CNP, O4 or MOG) at this stage, indicating that a majority of the OL differentiation and myelination occurred during the test compound treatment window (Additional file 9: Fig. S9). Using the microglial marker Iba1, we detected <1 % microglial cells at DIV5, and undetectable microglia at DIV13.

### NIH clinical collection library screening for compounds that promote myelination

To demonstrate that the assay conditions we developed were robust enough to support drug discovery screening efforts, we screened a small library of compounds. We selected the NIH Clinical Collection (NCC) library for screening which contains Food and Drug Administration (FDA)-approved off-patent drugs. Therefore, hits retrieved from this collection could lead to potential drug candidates for further development and rapid repositioning as therapeutics for MS. The NCC library consists of 727 biologically active compounds that have been through phase I–III clinical trials. This collection is additionally attractive because of the wide variety of cellular targets that are represented. Because this focused FDA-approved compound collection is small and the drug structures diverse, we decided to screen at two concentrations (5 and 1 μM) to reduce the possibility of missing hits due to false negatives. Each plate contained eight wells treated with DMSO or DAPT controls and each test compound concentration was screened in duplicate.

**Fig. 5:**
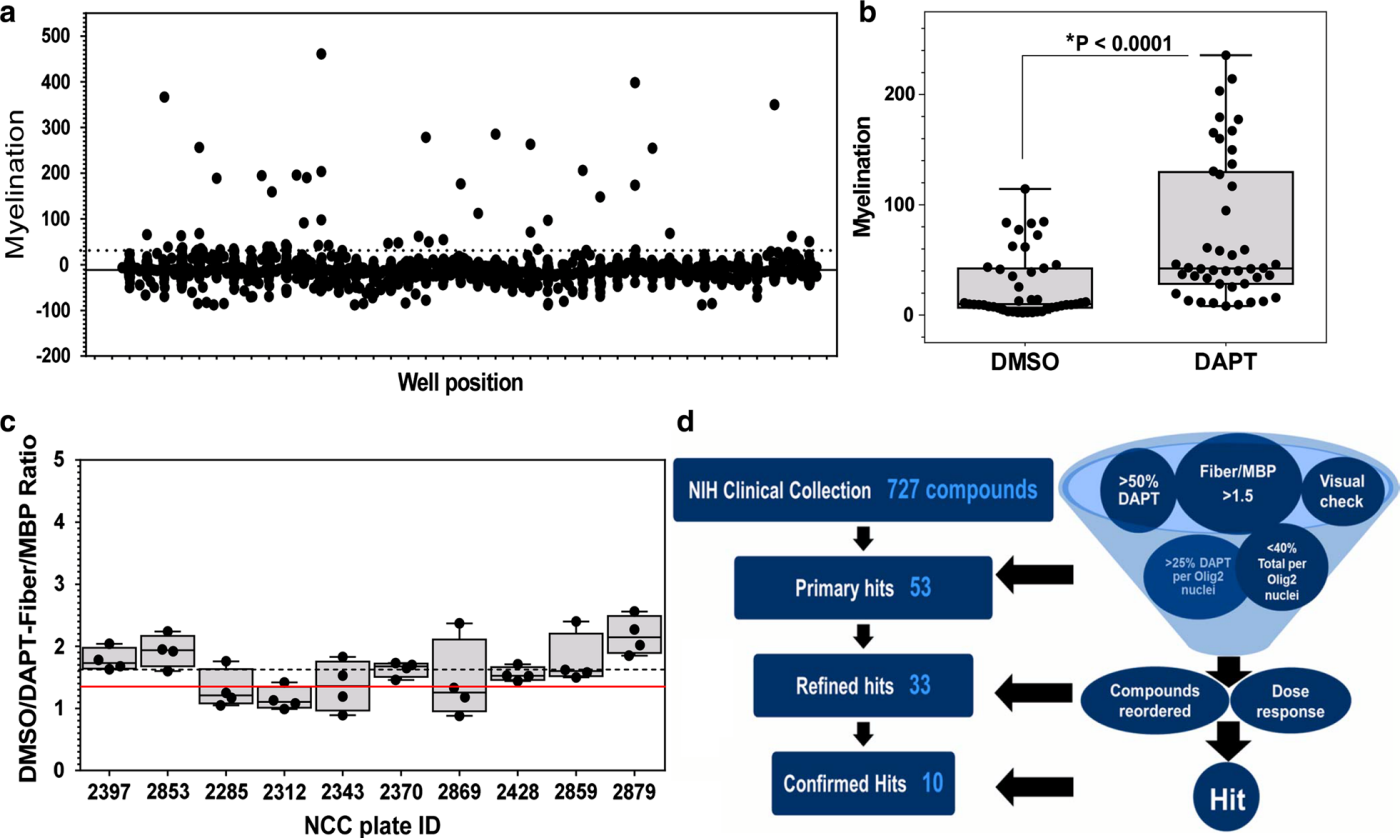
Analysis of the cortical myelination screen of the NCC compound library. **a** High-throughput screening data set used to identify promoters of myelination. The mean response is indicated by the *solid line*. The *dotted line* delineates the value of three SDs above the mean. Compounds that significantly reduced Olig2 expression were excluded. **b** High-throughput screening data plate control values of myelination. *Each point* is an compiled control value from each screening plate (n = 44, 16 image fields per concentration, mean ± SEM *P < 0.0001, *t* test). **c** Using the fiber score/MBP score as a specific measure of myelination (see Additional file 10: Fig. S10), the ratio of the DAPT to DMSO controls demonstrates the screening assay window. The *red line* delineates the cutoff value of 1.3. *Each point* is an averaged value from each screening plate (32 image fields per condition, mean ± SEM). The average DAPT/DMSO-fiber score/MBP score for the entire NCC library screen = 1.61 (*dashed line*). **d** NCC library hit selection process in the cortical culture myelination assay. Fifty-three primary hits compounds were initially identified from the NCC library with the criteria of >50 % DAPT and >1.5 fiber score/MBP score or fiber/MBP. The primary hits were further refined with additional criteria of >25 % DAPT/Olig2 nuclei ratio, <40 % DAPI/Olig2 nuclei ratio, and a visual morphology check to yield refined hits of 33 compounds. All refined hit compounds were reordered fresh and tested for efficacy in a dose–response profile. Ten compounds passed these criteria and were confirmed as hits

Automated image acquisition was performed from four randomized fields from each well, representing a total of eight data points per test concentration. The data was analyzed to find active compounds that increase myelin formation above a pre-defined threshold (>50 % of DAPT pro-myelinating activity). Though the threshold is somewhat arbitrary, we included in our analysis the delineation of three SDs above the mean signal of DMSO-treated well (Fig. 5a). While not a criterion in our assay for hit selection, it provided a statistical assurance that we were well out of a false-positive hit rate (0.15 %) range. Control DAPT versus DMSO values from the entire myelination screen were highly statistically significant (Fig. 5b) indicating an acceptable screen window.

We were concerned about the possibility of inter-preparation variability and differences in the behaviors of dissociated primary neurons and glia in culture, so we considered how to evaluate the consistency of responses to treatment in addition to assessing the consistency of the plate controls. As a measure of assay quality, we considered the Z-factor; however, since our assay is based on multiple readouts, we incorporated an additional parameter for assay window and robustness, a specific morphological measurement of early myelination. This criterion is a quotient of two morphological measurements—fiber length intensity score and MBP intensity score which adjusts for the contribution of OL differentiation (MBP expression) (Additional file 10: Fig. S10). A ratio of one indicates that an observed increase in myelination may almost be entirely accounted for by an increase in the extent of OL differentiation, whereas a value significantly greater than one indicates that there is an observed increase in myelination above and beyond what would be expected by an increase in OL differentiation alone, i.e. specific induction of myelination. We primarily used this to measure the quality of each plate in the screen. We typically observed DAPT treated cultures to have fiber score/MBP scores >1.5. Figure 5c depicts the DAPT/DMSO fiber score/MBP scores of each plate from the entire library screen which generated an average fiber score/MBP score of 1.61. For each plate in the screen, the acceptable fiber score/MBP score cutoff was in the range of 1.3 ± 0.2. In addition, the fiber score/MBP score was incorporated into the criteria for assay hits to potentially distinguish between active compounds with distinct mechanisms of action (see below). Additional file 12: Table S1 contains the data set for the entire library screen, showing the % DAPT normalized scores for myelination for each library compound.

### Selection criteria for myelination assay positive hit compounds

To select candidate compounds with substantial activity in our screen, compounds that had scores >50 % of DAPT were included as secondary hits (Fig. 5d). In addition, to further delineate the myelination effect enhanced by compounds, we implemented a second criterion of fiber score/MBP score (Additional file 10: Fig. S10) including any compounds that had scores >1.5 (fiber score/MBP scores for entire data set are included in Additional file 12: Table S1). Three compounds passed this criterion alone and were included in further hit compound refinement. We observed that some compounds displayed unusually large myelination scores but had very low overall Olig2^+^ nuclei expression, which could either reflect an inhibition of OL proliferation or increased OL cell death (e.g. methotrexate, Additional file 11: Fig. S11B7). Not surprisingly, visual assessment of images from these cultures revealed a low overall number of myelinating OLs. We therefore incorporated a third criterion to eliminate compounds that primarily act as anti-proliferative compounds and thus greatly reduced Olig2 expression: the ratio of total nuclei (DAPI staining)/Olig2^+^ nuclei. Large DAPI/Olig2^+^ nuclei numbers (>40) were an indicator that the test compound severely depleted OPCs and OLs, undesirable in a screen for compounds that promote myelination. DAPT reduces the number of Olig2^+^ cells by ~50 %, most likely by promoting OPC differentiation and reducing OPC proliferation [9]. Therefore, we implemented a criterion of >25 % of the DAPT Olig2^+^ cell count which also effectively eliminated compounds that severely reduced the number of Olig2^+^ cells (Fig. 5d). A fourth criterion was the qualitative assessment of OL MBP staining, taking into account the number of OLs/image field and OL morphology. Compounds that dramatically changed OL morphology (e.g. greatly enlarging the cell) while reducing the number of OLs/field were eliminated. Active compounds that passed all of these criteria were referred to as refined hits (Fig. 5d). A fifth criterion was to confirm activity and potency of refined hit compounds with full dose–response curve experiments of at least two replicates using reordered or resynthesized material. Actives that met this criterion were referred to as our confirmed hits (Fig. 5d) and our hit rate is based on this number. In a screen of 727 FDA-approved drugs, our screen identified 53 primary hits, 33 refined secondary hits and ten confirmed and reproducible hits (Table 1). The resulting hit rate for the entire screen was ~1.7 %. Additional file 11: Figure S11 shows the chemical structure of each hit compound, screening image of MBP/Olig2/DAPI staining, and the EC_50_ curves for myelination. Table 1 shows the calculated myelination EC_50_ values for the top hits from our cortical myelination screen. Based on the available literature on these previously characterized compounds, we grouped the hits based on the known mechanisms of action. These compounds fell into many different classes, grouped in Table 1, and are distinct from compounds previously identified by other library screens that have used OL differentiation assays in the absence of axons [2–4]. These data represent an initial screen to identify myelination-promoting compounds. Future studies will include an investigation of compound mechanisms as well as a thorough characterization of how these compounds effect cellular composition of the cultures.

## Discussion

### Development of high throughput cortical myelination assay

It has become clear that focusing solely on the immunological component of MS only addresses one aspect of the disease. Repairing damaged myelin and/or promoting the remyelination of demyelinated axons within lesions would, at a minimum, facilitate the preservation and/or restoration of some neuronal function. This may also prevent the irreversible neuronal damage believed to underlie the progressive disability that eventually affects most MS patients. Thus, remyelinating compounds are highly sought after, but have been difficult to identify in part because of the lack of HTS assays that truly detect myelination.

Our goal for developing a co-culture with live axons and oligodendrocytes as a myelinating in vitro system was to overcome the challenges of labor intensive OPC/neuron preparations, inconsistent performance of classical sources of neurons for modeling myelination (e.g. RGCs, dorsal root ganglion cells), and generating sufficient quantities of cells required for a robust HTS assay. In this study, we have described the development of the first in vitro myelination assay that assesses the functional dynamic interaction between live axons and oligodendrocytes during myelination and can be performed with a simple culture technique at a scale and reproducibility amenable to HTS drug discovery. This assay is unique in that it evaluates test compounds in the presence of the co-developing milieu of native brain cells, including OLs, neurons, and astrocytes. We demonstrate here that primary embryonic cortical tissue is an abundant cell source for both neurons and OPCs that are myelination competent [5, 6], easier to culture than RGCs, and widely used in large-scale HTS screening within the pharmaceutical industry. We have validated this assay using GSIs, establishing EC_50_ values for four different compounds to allow the ranking of potency. Using this assay as an initial screen, we screened the NCC library and identified ten confirmed hit compounds from diverse target classes for follow-up characterization.

Perhaps the most important aspects of the cortical myelination assay are that OLs develop and differentiate alongside growing axons and astrocytes, two major sources of signaling molecules known to influence myelination. The expression of the axonal protein LRR and Ig domain-containing, Nogo receptor-interacting protein (LINGO-1) was demonstrated be a potent inhibitor of differentiation and myelination [15–17]. Indeed, anti-LINGO-1 antibody is being developed as an MS therapeutic to promote axon remyelination and is currently in human clinical trials (BIIB033, ClinicalTrials.gov identifiers: NCT01244139, NCT01052506, NCT01864148). Leukemia inhibitory factor (LIF) has been shown to be released by astrocytes in response to ATP from action potential firing axons to promote myelination [16]. Additionally, through the action of TNFR2 on astrocytes, LIF is produced to stimulate OL differentiation in a co-culture system [18]. Furthermore, astrocytes were demonstrated to reduce OL differentiation, but specifically enhance myelin thickness and the rate of axon wrapping [9]. TNF impairs OL differentiation [19] attenuating TNF signaling by TNFR1 blocking therapy ameliorates MS symptoms in EAE [20]. It was also demonstrated that inhibiting glial γ-secretase promoted myelination [9], which also showed efficacy in vivo in the EAE model of MS [11]. These observations emphasize the importance of having culture conditions that more closely mimic the in vivo CNS composition.

It is important to note that this cortical myelination assay may fail to identify all possible compounds that may promote myelination. Due to the relatively short time course and single compound addition, our assay may not identify compounds with short half-lives, require multiple target activations, or require longer time courses. The assay could be modified by spiking in test compounds during the course of the ensheathment window and/or lengthen the ensheathment window longer than 8 days. We included T3, forskolin, and CNTF in the MyM medium as factors that facilitate OL differentiation and survival [21]. The activity of these factors may mask effects of potential stimulators of myelination. In particular, elimination of T3, may lower the threshold for identifying additional candidate compounds. This stimulation of differentiation by T3 may account for the lack of OL differentiation activity of benztropine and clemastine in our cortical myelination assay. Elimination of these factors from the myelination phase of the assay may reveal additional compounds with myelination activity.

### The cortical myelination assay identifies compounds not revealed by OL differentiation assays

Our myelination assay greatly differs from in vitro OL differentiation assays used for compound screening which have only assessed differentiation using purified OPCs (in isolation from axons and astrocytes) adapted to culture conditions by multiple passages [2, 3], or differentiated from induced pluripotent stem cells [22] and carried out in very short developmental time frames. Mei et al, 2014 [2] developed an HTS assay incorporating OL differentiation in the presence of inert micropillers allowing the quantification of pillar wrapping as a surrogate for myelination [2]. Lead compounds identified from these three studies, including clemastine, benztropine, miconazole and clobetasol, facilitate OL differentiation in cultures of purified OPCs [2–4] (Additional file 8: Fig. S8), but had no effect on myelination in our live axon myelination assay (Additional file 9: Fig. S9, Additional file 12: Table S1, compounds 450—clobetasol and 588—miconazole). Notably, our myelination assay did not identify muscarinic antagonists as previously identified by independent OL differentiation screens [2, 3], but revealed entirely new classes of compounds that promote myelination. No other high throughput assay has been developed to date capable of assessing the initiation and facilitation of myelination in the presence of axons, arguably the most important features when selecting candidate compounds capable of promoting or generating remyelination.

### Relation of cortical myelination assay hit compounds to clinical applications and multiple sclerosis

Repositioning approved drugs for the treatment of new indications is an activity that has grown in popularity in recent years and is a trend that is predicted to continue. There are many examples where repurposed drugs are making an impact in the field of MS. A few to note are BG-12 (Tecfidera^®^), a modification of Fumaderm^®^, used to treat psoriasis; fingolimod (Gilenya^®^), originally developed for transplantation and alemtuzumab (Lemtrada™), an oncology drug. We found that eight out of our ten confirmed hit compounds aligned with current MS repositioning efforts and are briefly cited here.

A treatment for congestive heart failure, digoxin (Lanoxin™) enhances both myelination and differentiation in our assay. It has been shown to reverse conduction block in demyelinated nerve fibers in experimental animals. Moreover, digoxin and possibly its derivatives, have also been implicated as a possible therapeutic for MS. A clinical study involving MS patients, showed improvement of symptoms when given the drug intravenously [23].

Other hits have shown efficacy in MS animal models. Imatinib mesylate (Gleevec™), a kinase inhibitor used for cancer treatment, is efficacious in experimental autoimmune encephalomyelitis (EAE) models [24]. Artesunate, a natural compound used to treat malaria, has a derivative form (artemisimin) that has anti-inflammatory properties in EAE [25]. Methotrexate (Trexall™), an anti-metabolite used to treat rheumatoid arthritis, is also efficacious at restoring myelin in cuprizone-induced demyelination mouse models [26]. In addition, methotrexate is commonly used off-label in MS, and is suggested as a low-dose, add-on therapy in combination with other treatments, to reduce the relapsing-remitting rate in MS [27] and to slow the progression of MS.

Some hits have been evaluated or will soon be tested in human MS patients. Oxcarbazepine (Trileptal^®^), a treatment for seizures and amilioride (Midamor^®^), a treatment for high blood pressure, have been demonstrated to be neuroprotective in rodent models of MS and in MS patients [28], and are now in human clinical trials to treat progressive MS (PROXIMUS Trial NCT02104661; MS-SMART Trial NCT01910259). Additionally, there is a patent for docetaxel (Taxotere^®^), an anti-microtubule and treatment for cancer (US6515016B2) on its use to treat or prevent psoriasis and MS.

Off-label muscle relaxants are used, when necessary, to help MS patients offset or reduce relapsing-remitting episodes. Atracurium besylate (Tracrium™) is used as an adjunct to general anesthesia and particularly useful for MS patients undergoing surgery since general anesthesia can exacerbate MS symptoms. In a clinical case report, a patient with MS underwent surgery for renal failure, and in a 3 month follow-up, reported no further MS attacks [29].

The remainder of our confirmed hits, zu-capsaicin (Civanex™) and tegafur (Uftoral^®^) have not been tested in any demyelinating diseases.

To our knowledge the myelin restorative effects of these compounds have not been evaluated. Our data suggests that these drugs should additionally focus on testing these hits to determine whether they have beneficial effects on remyelination in vivo and possibly in MS patients.

## Conclusion

We have developed the first high capacity, phenotypic in vitro assay amenable to high throughput screening to identify compounds that promote myelination. Our myelination assay incorporates both live axons and astrocytes, both of which have profound effects on myelination in vivo. Our assay utilizes primary cortical cells under conditions that allows for endogenous myelination, and thus avoids potential artefacts due to the use of cell lines or artificial substrates for myelination. To demonstrate the utility of this assay, we have screened the NCC library of FDA-approved compounds, identifying ten compounds for follow up studies, including effects on different cell types, mechanisms of action, structure–activity relationship determination, and in in vivo models of MS. Our hope is that this cortical myelination assay will be scaled up to screen even larger compound libraries in search of more efficacious MS therapeutics.

## Methods

### Reagents

Dulbecco’s modified Eagle Medium (DMEM) high glucose, Neurobasal medium (NB), Hank’s balanced Salt Solution (HBSS), Earle’s balanced Salt Solution, l-glutamine, sodium pyruvate, penicillin/streptomycin, Diamidino-2-Phenylindole, Dilactate (DAPI) were purchased from Life Technologies (Carlsbad, CA, USA), N21-MAX medium supplement from R&D Systems (Minneapolis, MN, USA), normal goat and fetal bovine serum, forskolin, triiodothyronine (T3), vitamin B12, hydrocortisone, biotin, boric acid, apotransferrin, putrescine, progesterone, sodium selenite, poly-d-lysine, recombinant human insulin, bovine serum albumin and DMSO were obtained from Sigma-Aldrich (St. Louis, MO, USA). Trace elements B and trypsin 0.05 %-EDTA were purchased from Mediatech, Inc. (Manassas, VA, USA). Human ceruloplasmin was purchased from EMD Millipore (Billerica, MA, USA). Recombinant human BDNF and CNTF were purchased from PeproTech (Rock Hill, NJ, USA). Laminin was obtained from Trevigen (Gaithersburg, MD, USA). DNase and papain were purchased from Worthington Biochemical Corporation (Lakewood, NJ, USA). Packard Viewplates 96-well were purchased from Perkin Elmer (Waltham, MA, USA). Additional file 13: Table S2 lists the primary antibodies and their dilutions used in this study.

### Cell culture methods

All animal work was carried out in strict accordance with the recommendations in the Guide for the Care and Use of Laboratory Animals of the National Institutes of Health. All animal protocols were approved by Institutional Animal Care and Use Committee (IACUC) at the Molecular Medicine Research Institute. Animals were either euthanized by CO_2_ asphyxiation or decapitation.

### RGC-OPC culture methods

RGCs were prepared from P6-P7 Sprague–Dawley rat pups (Charles River, Wilmington, MA, USA), following the RGC immunopanning purification protocol as described in Watkins et al. [9]. On DIV11 of RGC culture, cortical OPCs were purified from P7 Sprague–Dawley rat pups, following the OPC immunopanning purification protocol (as described in [30]). Six days following test compound addition (17 DIV), cells were fixed, immunostained and imaged as described below.

### Embryonic cortical culture methods

The dissection of E18 rat (Charles River, Wilmington, MA, USA) cortex is similar to that described previously [31–33] with some modifications. Briefly whole cortices from three embryos were collected in a petri dish containing HBSS. After carefully removing the meninges, the tissue was divided into cortical hemispheres, dissected and the non-cortical structures were removed. Cortical tissue was then digested in 7 U/ml papain dissolved in HBSS with 500 U/ml DNase I, and incubated for 30 min at 35 °C. The enzymatic reaction was terminated with DMEM containing 10 % FBS. The tissue was allowed to settle, supernatant was removed and tissue was triturated with a flame-polished glass Pasteur pipette in DMEM/10 % FBS, 250 U/ml DNAse I until the tissue was completely dispersed. The dissociated cell suspension was centrifuged at 200×*g* for 5 min and supernatant replaced with plating medium (NB medium with 1× N21 supplement and 2 mM l-glutamine and 1 % penicillin–streptomycin). Viable cells were counted using trypan blue exclusion and typically exceeded 80 %. Isolated cells were seeded onto 96-well plates pre-coated with poly-d-lysine (10 μg/ml) and laminin (2 μg/ml) at a density of 20,000 cells/well (2 × 10^5^ cell/cm^3^). Neurons were allowed to adhere, recover, mature and extend axons for 3 days. On the fourth day, the plating medium was diluted with an equivolume of myelination medium (MyM), as described in Watkins et al. [9] with minor modifications (see “Results”). The following day, two-thirds of the medium was replaced with fresh MyM and test compound. The day after establishing the primary culture was defined as day 1 in vitro (DIV1).

### Acute oligodendrocyte differentiation assay

OPCs from P7 Sprague–Dawley rat pups were purified by immunopanning and cultured as described [30]. OPCs were plated at 5000 cells/well into pDL-Laminin coated 96-well TC plate wells and centrifuged at 200×*g* to facilitate cell attachment, survival, and assure even distribution of OPCs. Plated OPCs were pre-incubated for 1–2 h at 37 °C in 10 % CO_2_ incubator, followed by addition of test compounds in quadruplicate. Controls were added in eight replicate wells, negative control = 0.1 % DMSO final concentration; positive control = 40 ng/ml T3. The day of OPC plating was considered DIV0. On DIV4, cells were fixed, immunostained, and imaged as described below. Minor modifications include blocking cells with 10 % normal goat serum (NGS)/0.4 % Triton X-100 and staining overnight at 4 °C with rat anti-MBP antibodies diluted in 10 % NGS/0.08 % Triton X-100. OL differentiation was quantified by IN Cell Developer Toolbox image analysis software which calculated the MBP staining intensity of two images per well. The extent of OL differentiation was defined by the total threshold-selected area of MBP staining x MBP fluorescence intensity in this area divided by the total number of OLs (identified by DAPI nuclear staining).

### Immunofluorescence staining and imaging

At the experimental end point, medium was removed leaving 50 μl/well using an ELX405 microplate washer (BioTek, Winooski, VT, USA). Cells were then fixed for 14 min with paraformaldehyde solution to a final concentration of 4 %. Following fixation, plates were washed with 1 ml PBS leaving 50 μl/well using the microplate washer. Cells were then blocked in blocking buffer (10 % normal goat serum, 0.1 % Triton X-100), antibody buffer (150 mM NaCl, 50 mM Tris Base, 1 % BSA, 100 mM l-lysine, 0.004 % sodium azide, pH 7.4), and stained with mouse anti-rat MBP antibody and anti-rabbit Olig2 diluted in blocking buffer overnight at 4 °C. The cells were washed and incubated with secondary antibodies, and DAPI, 0.3 μM for 1 h at room temperature. After a final wash, 100 μl of PBS was added to each well and plates imaged. Images were captured with a Nikon Eclipse TE-2000-U microscope, Zyla cMOS megapixel camera (ANDOR Technology, Belfast, UK), fitted with an automated stage controlled by NIS Elements AR software 4.0 (Melville, NY, USA). An air 10X lens was used to capture four images per well with 16 bit resolution, 2560 × 2160 pixels. Images for each assay run were captured using identical camera settings. Images were exported as TIFF files for analysis and quantification.

### Image quantification

TIFF files were analyzed using a custom algorithm created with IN Cell Investigator Developer Toolbox (GE Health Sciences, Piscataway, NJ, USA). For each well, four images were analyzed and the data from the duplicate well combined and averaged (total of eight images per test condition). The extent of OL differentiation was defined by the total threshold-selected area of MBP staining × MBP fluorescence intensity in this area divided by the total number of OLs (identified by Olig2 nuclear staining). We referred to this as the “MBP score” or “OL differentiation”. Earlier publications have characterized in vitro myelination as contiguous segments of MBP staining co-localizing with axons, representing the contact and ensheathment of axons with the myelin membrane generated by OLs. Hence, our assay defined myelination as the alignment of MBP staining assuming the shape change into long straight contiguous segments. Our fiber length algorithm delineated only what it identified as continuous straight lines from an image field, and then applied morphometric quantification of intensity of those selected lines (calculates the total pixel length within a single fibrous shape). This value was then divided by the total number of OLs to give the value referred to as “fiber score” or “myelination”. The quotient of the myelination score and the MBP score equals a value we referred to as “fiber score/MBP score”, reflecting myelination independent of the effects of differentiation; in other words, MBP staining specific to myelination. Numerical results from the analyzed images were later exported for analysis in Microsoft Excel (Redmond, WA, USA). Data was normalized by fitting parameters to positive (1 μM DAPT) and negative controls (0.1 % DMSO) and expressed as the % of DAPT.

### Relative EC_50_ analysis

Half maximal effect concentrations (EC_50_) values were obtained by fitting the data to a sigmoidal dose–response curve-fitting function (Prism, GraphPad software, La Jolla, CA, USA). Serial dilutions of eight to ten different concentrations with four data points per concentration were used for curve fitting. Experiments were repeated at least two times.

### Compounds

All compounds in the NCC library were supplied in DMSO at 10 mM in 96-well plates. Hit compounds were purchased as powders and stocks dissolved in DMSO to 10 mM for in vitro studies (see Additional file 13: Table S2 for complete listing of compounds). *N*-[*N*-(3,5-Difluorophenacetyl)-l-alanyl]-*S*-phenylglycine t-butyl ester (DAPT), LY411,575, and BMS 708163 were from Selleckchem, MRK560 was purchased from Tocris.

### Statistical methods

For all experiments, assuming normal distribution, two-tailed t-tests were used to evaluate comparisons between two groups and ANOVA was used when more than two groups were compared. For the quantitative analysis of in vitro myelination and differentiation, a one-way ANOVA was conducted to compare the effect of each dose of compound with DMSO (Prism, GraphPad software, La Jolla, CA, USA). This was followed by post hoc comparisons using Bonferroni or Dunnett Multiple comparison tests. Where possible, data were represented as the mean (M) ± standard error of the mean (SEM) or standard deviation (SD) unless otherwise indicated in the figure legends.

## Additional files

10.1186/s12868-016-0250-2 Determination of embryonic cortical cultures for screening suitability.
10.1186/s12868-016-0250-2 The addition of exogenous OPCs to embryonic cortical cultures is not required for quantitative myelination.
10.1186/s12868-016-0250-2 Determination of optimal time courses for myelination in the cortical cell myelination assay.
10.1186/s12868-016-0250-2 γ–secretase inhibitors do not promote OL differentiation, whereas benztropine and clemastine facilitate OL differentiation in an OL differentiation assay with acutely purified OPCs.
10.1186/s12868-016-0250-2 Benztropine and clemastine show little to no activity in the cortical myelination assay.
10.1186/s12868-016-0250-2 Neuronal characterization of DIV13 cortical cultures.
10.1186/s12868-016-0250-2 Neuronal characterization of DIV5 cortical cultures.
10.1186/s12868-016-0250-2 A2B5 marker antibodies identify abundant glial progenitor cells in DIV5 cortical cultures, but are largely absent in DIV13 cultures.
10.1186/s12868-016-0250-2 Oligodendrocyte characterization of DIV5 and DIV13 cortical cultures, demonstrate robust OL differentiation during the test compound treatment window.
10.1186/s12868-016-0250-2 Equations for the quantification of myelination.
10.1186/s12868-016-0250-2 Structures, images, and EC_50_ curves of cortical myelination and OL differentiation hits.
10.1186/s12868-016-0250-2 NCC library screen data set.
10.1186/s12868-016-0250-2 Antibodies.

## Abbreviations

CNS: central nervous system
MS: multiple sclerosis
OL: oligodendrocytes
OPCs: oligodendrocyte precursor cells
DIV: days in vitro
RGC: retinal ganglion cell
GSI: γ-secretase inhibitor
MBP: myelin basic protein
DAPT: *N*-[*N*-(3,5-difluorophenacetyl)-l-alanyl]-*S*-phenylglycine t-butyl ester
DMSO: dimethylsulfoxide
HTS: high throughput screening
NCC: NIH clinical collection
