# Supplementary material for: Development of a central nervous system axonal myelination assay for high throughput screening
Source: BMC Neurosci. 2016 Apr 22;17:16. doi: 10.1186/s12868-016-0250-2 (PMC4840960; doi:10.1186/s12868-016-0250-2)
Supplement: Supplementary file 1 — 10.1186/s12868-016-0250-2 Determination of embryonic cortical cultures for screening suitability. [file 12868_2016_250_MOESM1_ESM.pdf]

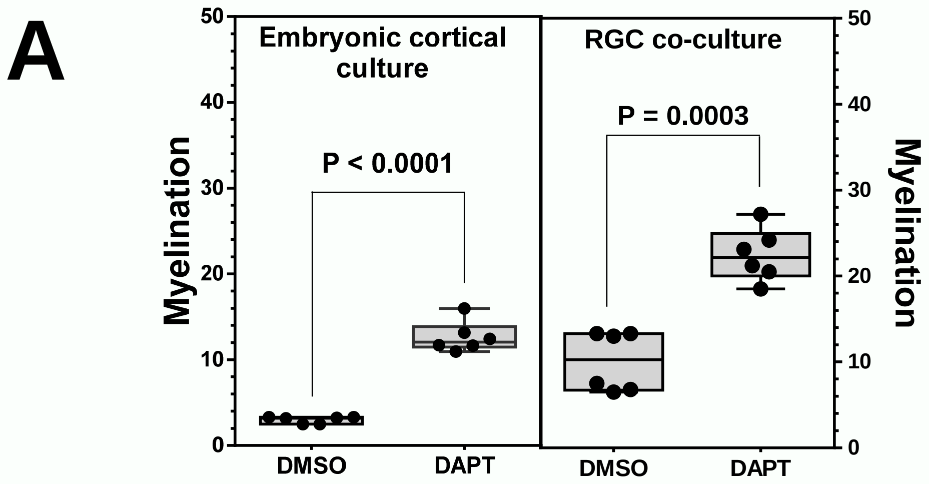

	DMSO	DAPT
CV	13.14%	14.24%

	DMSO	DAPT
CV	34.27%	13.66%

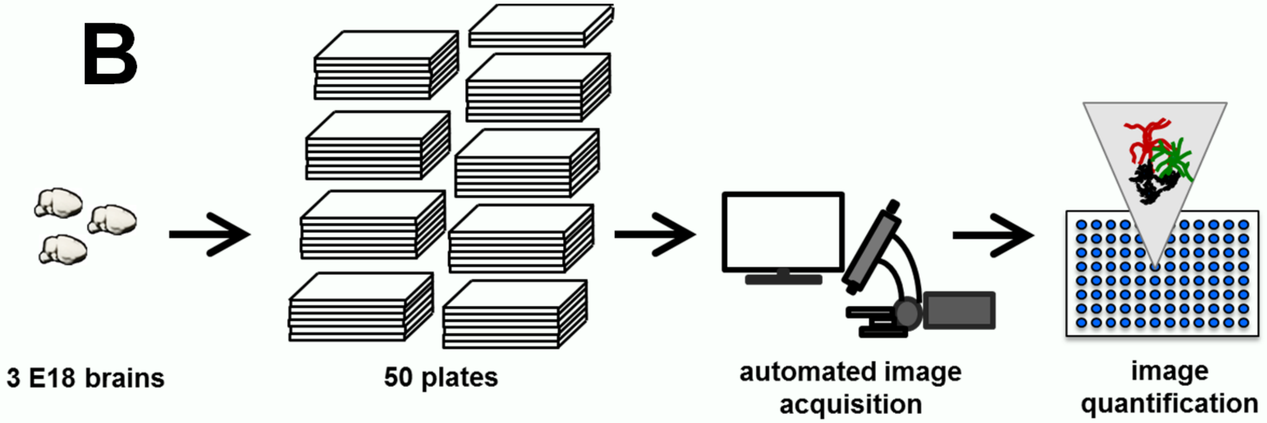

Figure S1. Determination of embryonic cortical cultures for screening suitability. A, Myelination quantification of DMSO and DAPT control values were compared in two types of myelination culture preparations. Data shown was compiled from n = 6 experiments, 32 image fields per test condition, mean ± SEM. P values versus DMSO were determined by two-tailed *t*-test. Coefficient of variation (CV) values are reported below the graphs. CV values <20% were considered in the acceptable range. B, Schematic of the cortical co-culture preparation that demonstrates that three embryonic brains used for the cortical co-culture myelination assay will yield approximately fifty 96-well plates.
