# Supplementary material for: Development of a central nervous system axonal myelination assay for high throughput screening
Source: BMC Neurosci. 2016 Apr 22;17:16. doi: 10.1186/s12868-016-0250-2 (PMC4840960; doi:10.1186/s12868-016-0250-2)
Supplement: Supplementary file 2 — 10.1186/s12868-016-0250-2 The addition of exogenous OPCs to embryonic cortical cultures is not required for quantitative myelination. [file 12868_2016_250_MOESM2_ESM.pdf]

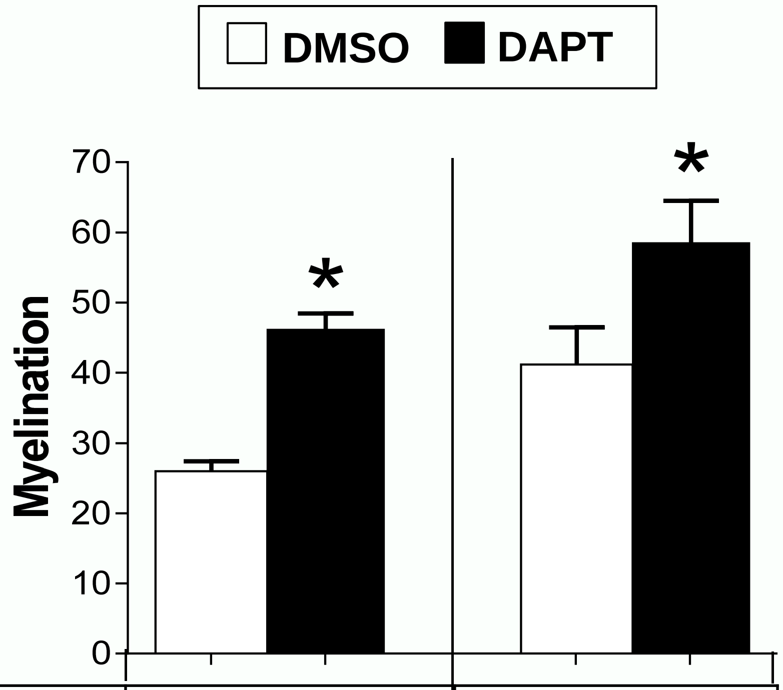

	No OPCs added		OPCs added	
**DAPT/DMSO ratio**	**1.76**		**1.14**	
**Mean**	25.97	45.80	41.18	58.14
**SD**	5.77	10.54	21.16	25.52
**SEM**	1.44	2.64	5.29	6.38
**CV**	22.23%	23.02%	51.38%	43.89%

Figure S2. The addition of exogenous OPCs to embryonic cortical cultures is not required for quantitative myelination. The promotion of myelination with DAPT was more robust (1.76 fold over DMSO) in cultures without exogenously added OPCs. The asterisk (*) denotes P values versus DMSO of < 0.0001, *t*-test. A table of mean, standard deviation (SD), standard error of mean (SEM) and coefficient of variation (CV) values are reported below columns (64 image fields per treatment, mean ± SEM). CV values of ≤20% ± 5% were considered in the acceptable range.
