# Supplementary material for: Development of a central nervous system axonal myelination assay for high throughput screening
Source: BMC Neurosci. 2016 Apr 22;17:16. doi: 10.1186/s12868-016-0250-2 (PMC4840960; doi:10.1186/s12868-016-0250-2)
Supplement: Supplementary file 3 — 10.1186/s12868-016-0250-2 Determination of optimal time courses for myelination in the cortical cell myelination assay. [file 12868_2016_250_MOESM3_ESM.pdf]

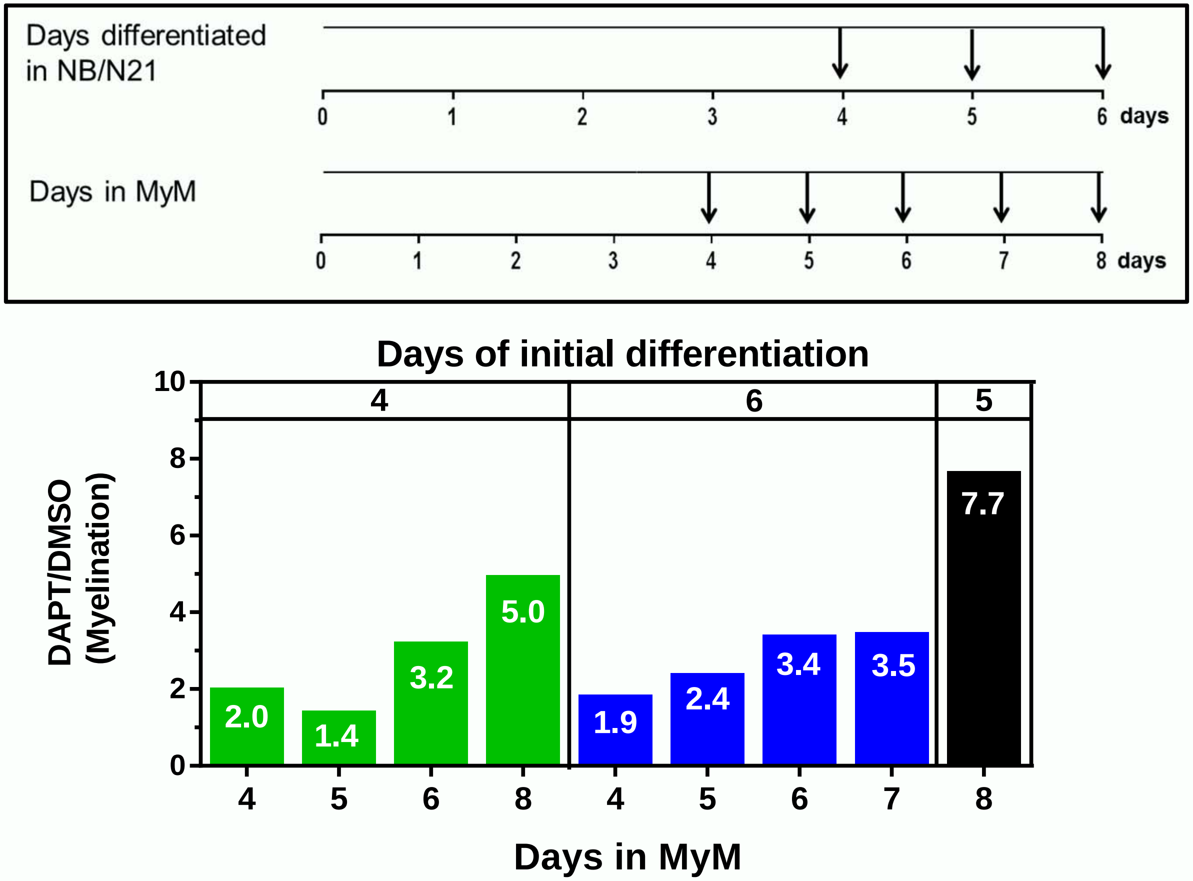

Figure S3. Determination of optimal time courses for myelination in the cortical cell myelination assay. E18 cortical cultures were initially differentiated for either 4 days (green bars), 5 days (black bars), or 6 days (blue bars) in NB/N21 media, followed by 4, 5, 6, 7, or 8 days in MyM, then fixed for antibody staining and image analysis. Numbers in bars indicate the DAPT/DMSO myelination ratio for each condition. The ratio values were compiled from 64 image fields, mean ± SEM. The time course with greatest DAPT/DMSO myelination ratio was 5 days NB/N21 and 8 days MyM plus test compound (ratio = 7.7) and was used in all subsequent assay development and screening.
