# Supplementary material for: Development of a central nervous system axonal myelination assay for high throughput screening
Source: BMC Neurosci. 2016 Apr 22;17:16. doi: 10.1186/s12868-016-0250-2 (PMC4840960; doi:10.1186/s12868-016-0250-2)
Supplement: Supplementary file 4 — 10.1186/s12868-016-0250-2 γ–secretase inhibitors do not promote OL differentiation, whereas benztropine and clemastine facilitate OL differentiation in an OL differentiation assay with acutely purified OPCs. [file 12868_2016_250_MOESM4_ESM.pdf]

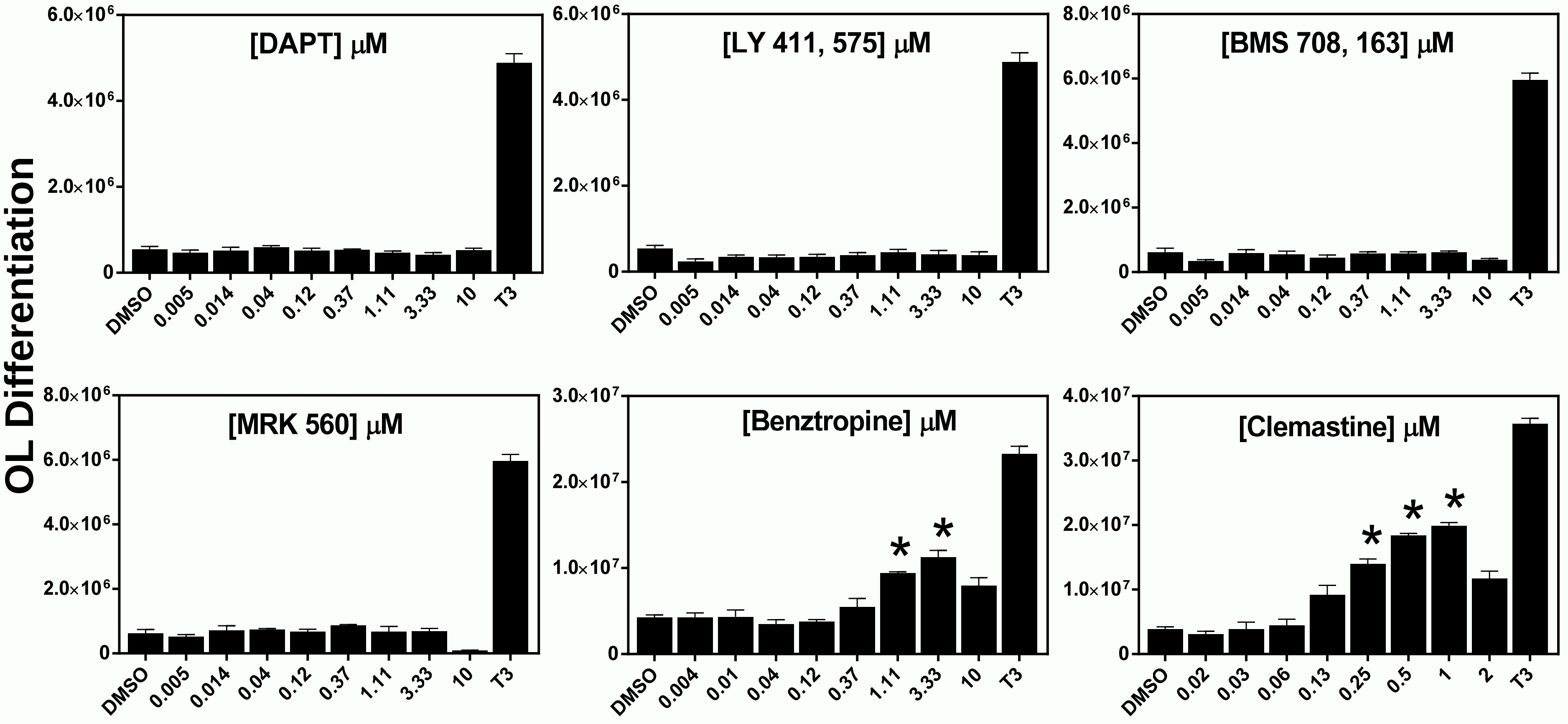

Figure S4. γ–secretase inhibitors do not promote OL differentiation, whereas benztropine and clemastine facilitate OL differentiation in an OL differentiation assay with acutely purified OPCs. Acutely prepared OPCs were cultured for 4 days (see methods) in the presence of increasing concentrations of test compound. 0.1% DMSO and 40 ng/ml T3 serve as negative and positive controls, respectively. Representative data shown are averaged from eight image fields per test concentration, mean ± SEM. * denotes P values versus DMSO of < 0.0001, ANOVA with Bonferroni post hoc test. For benztropine, there was a significant effect of two compound concentrations compared to DMSO [$F(2.25, 6.75) = 77.80$, $p < 0.0001$]. Post hoc comparisons indicated that the mean score for the concentrations 1.11 μM ($M = 9.4x10^6$, $SEM = 1.71x10^5$), 3.33 μM ($M = 1.13x10^7$, $SEM = 8.01x10^5$), was significantly different than DMSO. For clemastine, there was a significant effect of three compound concentrations compared to DMSO [$F(10, 3) = 106.9$, $p < 0.0001$]. Post hoc comparisons using the Bonferroni test indicated that the mean score for the concentrations 0.25 μM ($M = 1.4x10^7$, $SEM = 7.88x10^5$), 0.5 μM ($M = 1.84x10^7$, $SEM = 3.37x10^5$), 1 μM ($M = 1.99x10^7$, $SEM = 4.7x10^5$) was significantly different than DMSO.
