# Supplementary material for: Development of a central nervous system axonal myelination assay for high throughput screening
Source: BMC Neurosci. 2016 Apr 22;17:16. doi: 10.1186/s12868-016-0250-2 (PMC4840960; doi:10.1186/s12868-016-0250-2)
Supplement: Supplementary file 5 — 10.1186/s12868-016-0250-2 Benztropine and clemastine show little to no activity in the cortical myelination assay. [file 12868_2016_250_MOESM5_ESM.pdf]

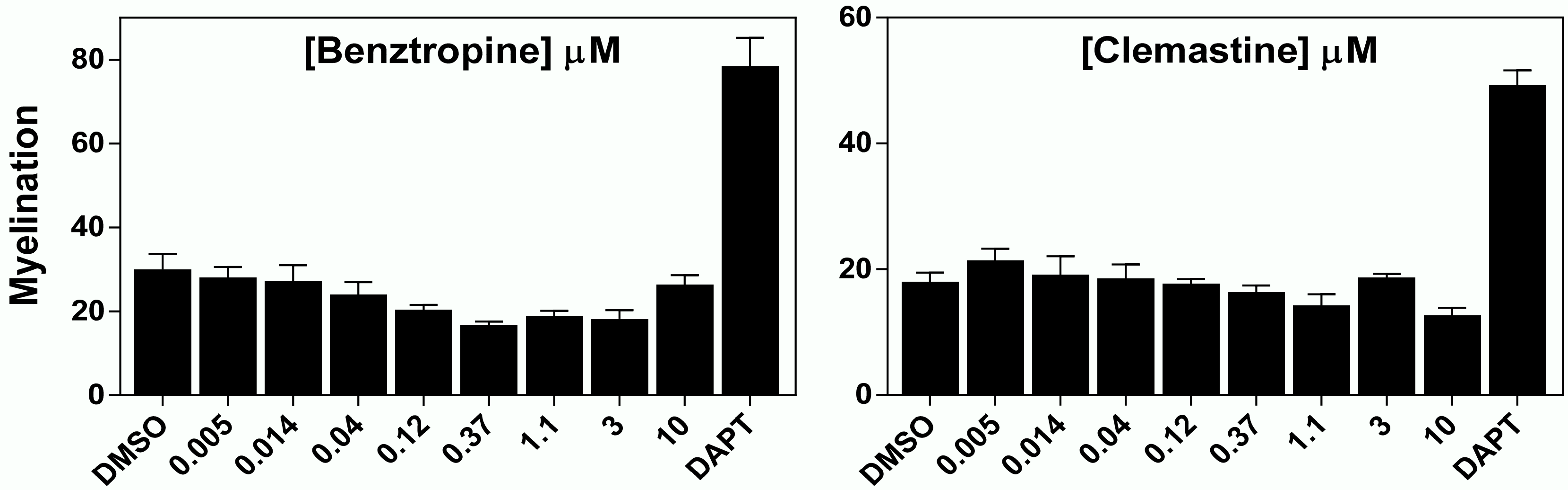

Figure S5. Benztropine and clemastine show little to no activity in the cortical myelination assay. Dose response experiments were performed adding test compound to cortical cultures on DIV5 and incubated for an additional eight days as described above. Representative raw data is averaged from 16 image fields per concentration, mean ± SEM.
