# Supplementary material for: Development of a central nervous system axonal myelination assay for high throughput screening
Source: BMC Neurosci. 2016 Apr 22;17:16. doi: 10.1186/s12868-016-0250-2 (PMC4840960; doi:10.1186/s12868-016-0250-2)
Supplement: Supplementary file 6 — 10.1186/s12868-016-0250-2 Neuronal characterization of DIV13 cortical cultures. [file 12868_2016_250_MOESM6_ESM.pdf]

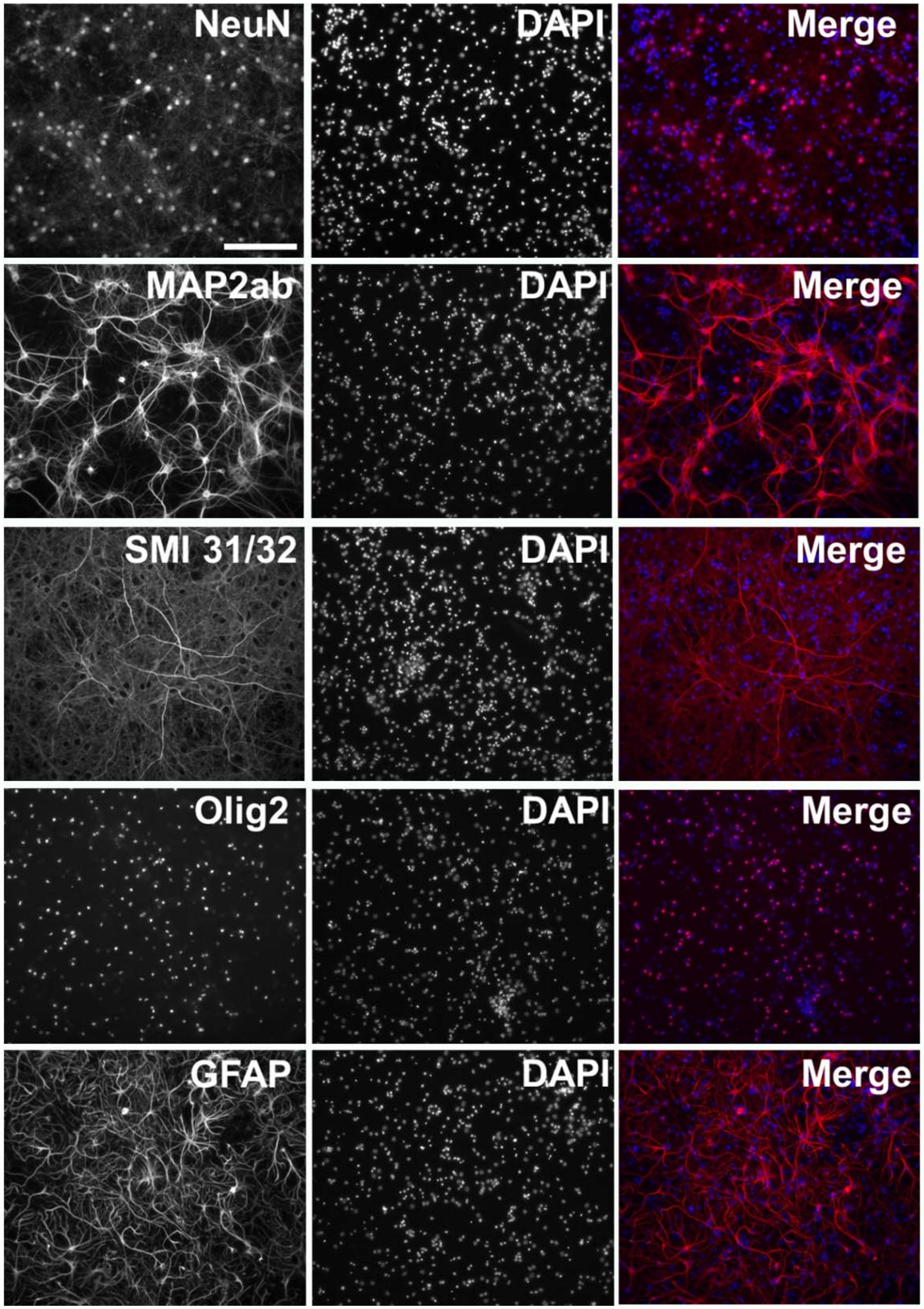

Figure S6. Neuronal characterization of DIV13 cortical cultures. Control cortical cultures were treated with 0.1% DMSO on DIV5, then on DIV13, fixed and stained with the antibodies labeled in the left panels. Right images are merged from left and middle panels with antibody staining in red and DAPI staining in blue, overlapping staining appears pink. Counting NeuN, Olig2, and GFAP positive cells with overlapping DAPI staining, these cultures were calculated to have approximately 22.5% neurons, 22% OPCs/OLs, and 46% astrocytes. Bar = 200 µm.
