# Supplementary material for: Development of a central nervous system axonal myelination assay for high throughput screening
Source: BMC Neurosci. 2016 Apr 22;17:16. doi: 10.1186/s12868-016-0250-2 (PMC4840960; doi:10.1186/s12868-016-0250-2)
Supplement: Supplementary file 7 — 10.1186/s12868-016-0250-2 Neuronal characterization of DIV5 cortical cultures. [file 12868_2016_250_MOESM7_ESM.pdf]

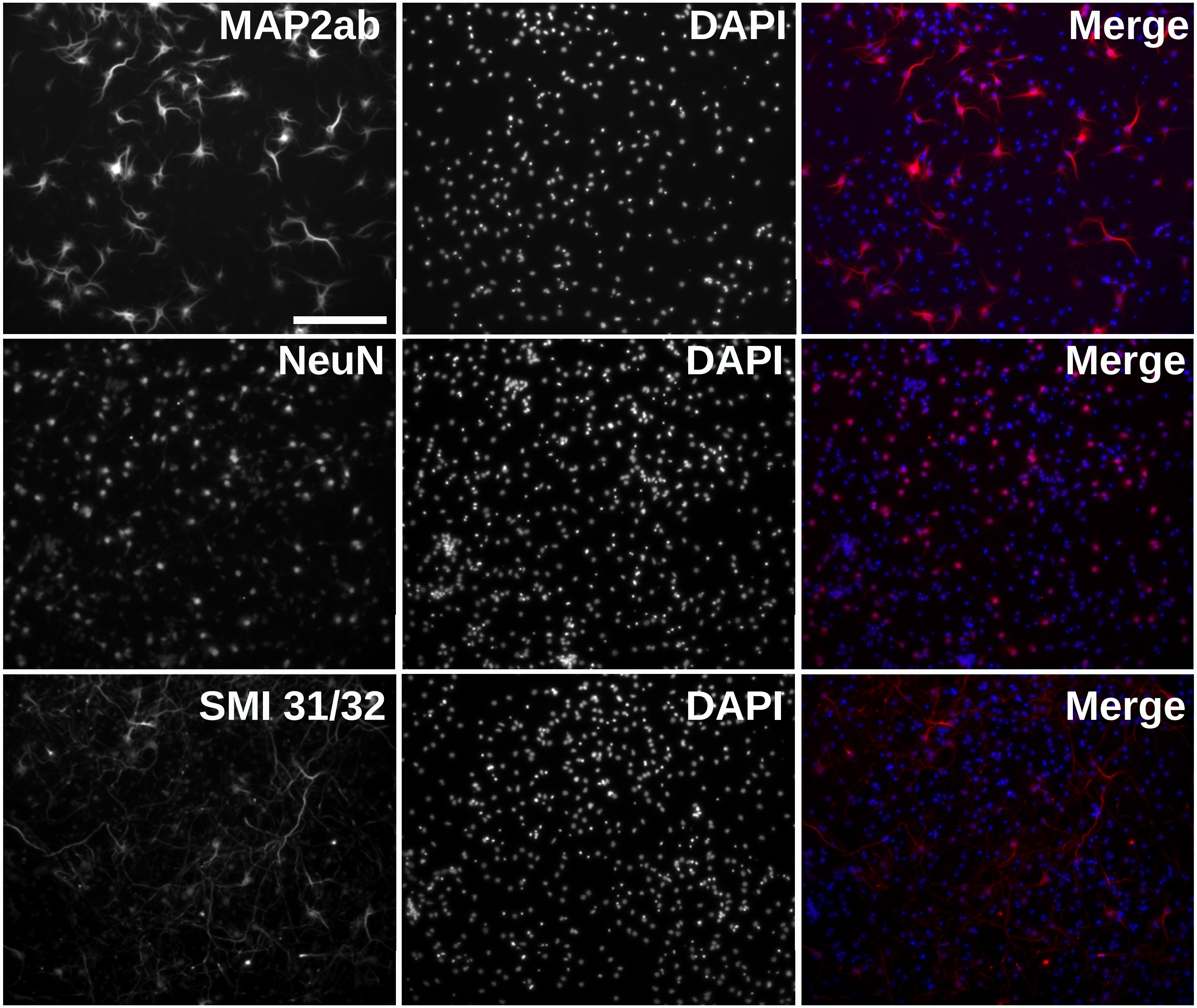

Figure S7. Neuronal characterization of DIV5 cortical cultures. Cortical cultures were established and grown until DIV5, fixed and stained with the antibodies labeled in the left panels. Right images are merged from left and middle panels with antibody staining in red and DAPI staining in blue, overlapping staining appears pink. Bar = 200 µm.
