# Supplementary material for: Development of a central nervous system axonal myelination assay for high throughput screening
Source: BMC Neurosci. 2016 Apr 22;17:16. doi: 10.1186/s12868-016-0250-2 (PMC4840960; doi:10.1186/s12868-016-0250-2)
Supplement: Supplementary file 8 — 10.1186/s12868-016-0250-2 A2B5 marker antibodies identify abundant glial progenitor cells in DIV5 cortical cultures, but are largely absent in DIV13 cultures. [file 12868_2016_250_MOESM8_ESM.pdf]

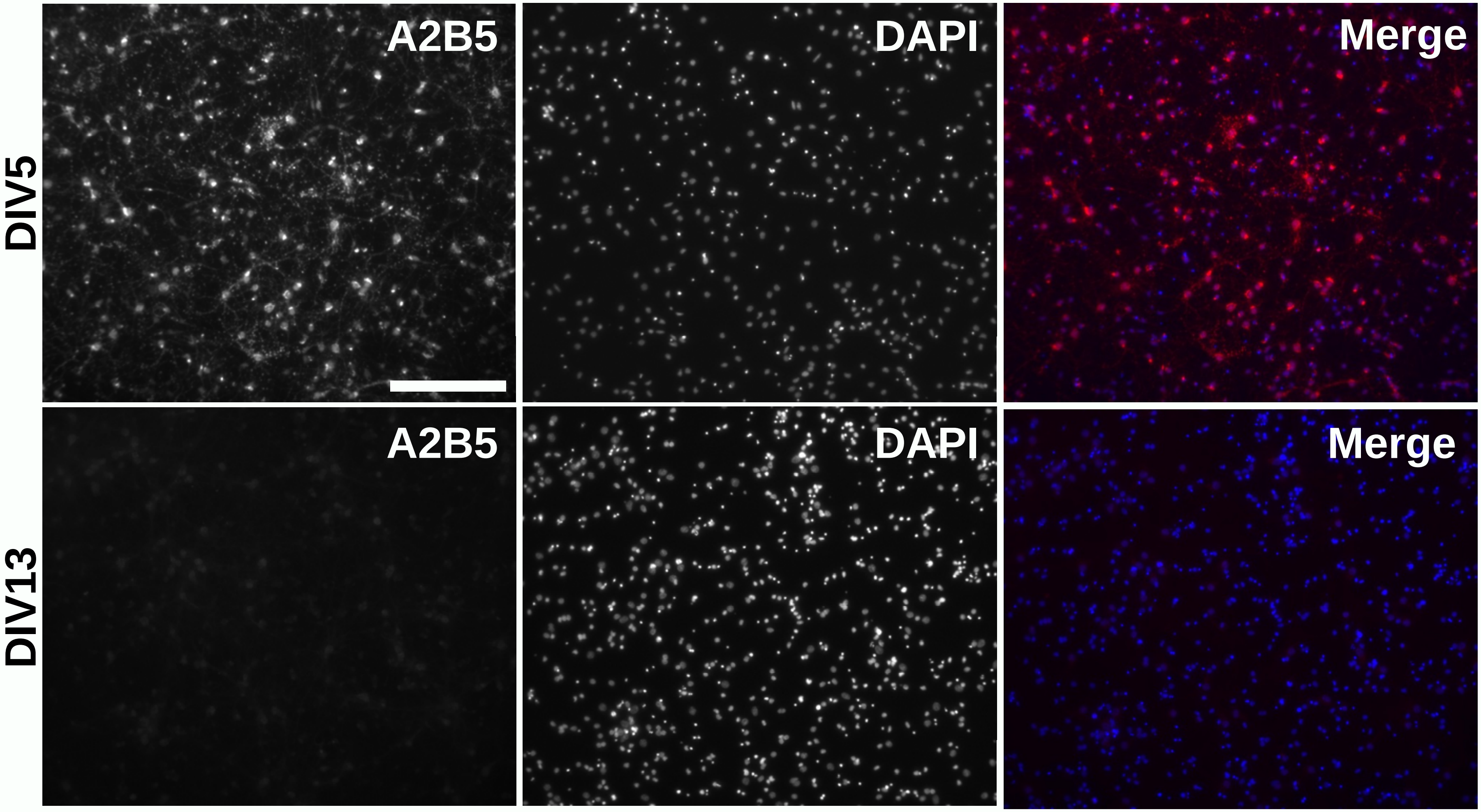

Figure S8. A2B5 marker antibodies identify abundant glial progenitor cells in DIV5 cortical cultures, but are largely absent in DIV13 cultures. Cortical cultures were grown, fixed and stained with anti-A2B5 antibodies on either DIV5 (day of test compound addition) or DIV13 (endpoint of myelination assay). Images at the right show the merged images of A2B5 (red) and DAPI (blue). Note the almost complete absence of A2B5 staining in the DIV13 cultures. Bar = 200 µm.
