# Supplementary material for: Development of a central nervous system axonal myelination assay for high throughput screening
Source: BMC Neurosci. 2016 Apr 22;17:16. doi: 10.1186/s12868-016-0250-2 (PMC4840960; doi:10.1186/s12868-016-0250-2)
Supplement: Supplementary file 9 — 10.1186/s12868-016-0250-2 Oligodendrocyte characterization of DIV5 and DIV13 cortical cultures, demonstrate robust OL differentiation during the test compound treatment window. [file 12868_2016_250_MOESM9_ESM.pdf]

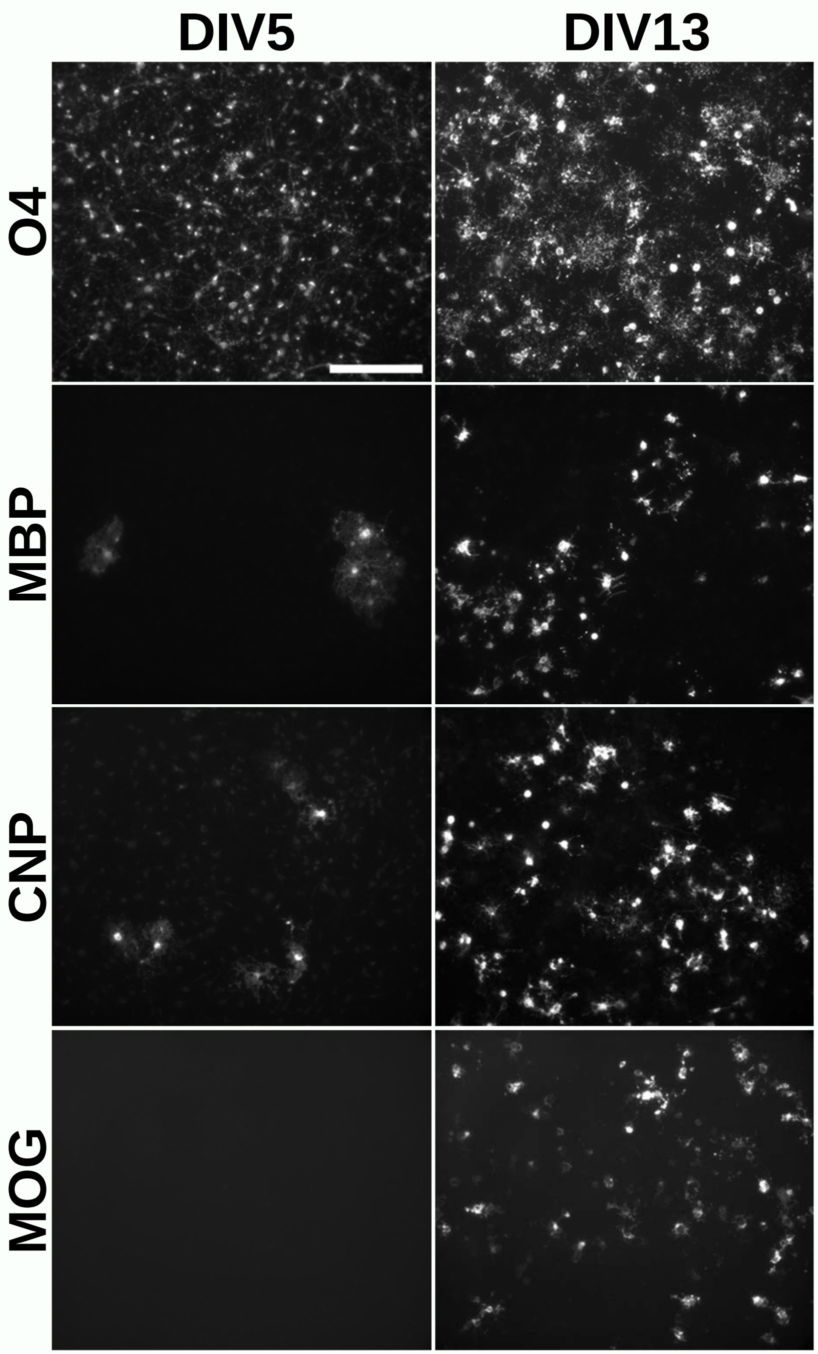

Figure S9. Oligodendrocyte characterization of DIV5 and DIV13 cortical cultures, demonstrate robust OL differentiation during the test compound treatment window. Cortical cultures were grown, fixed and stained with the antibodies labeled at the left. Note the robust expression of OL markers in the DIV13 cultures. Bar = 200 µm.
