# Supplementary material for: Development of a central nervous system axonal myelination assay for high throughput screening
Source: BMC Neurosci. 2016 Apr 22;17:16. doi: 10.1186/s12868-016-0250-2 (PMC4840960; doi:10.1186/s12868-016-0250-2)
Supplement: Supplementary file 10 — 10.1186/s12868-016-0250-2 Equations for the quantification of myelination. [file 12868_2016_250_MOESM10_ESM.pdf]

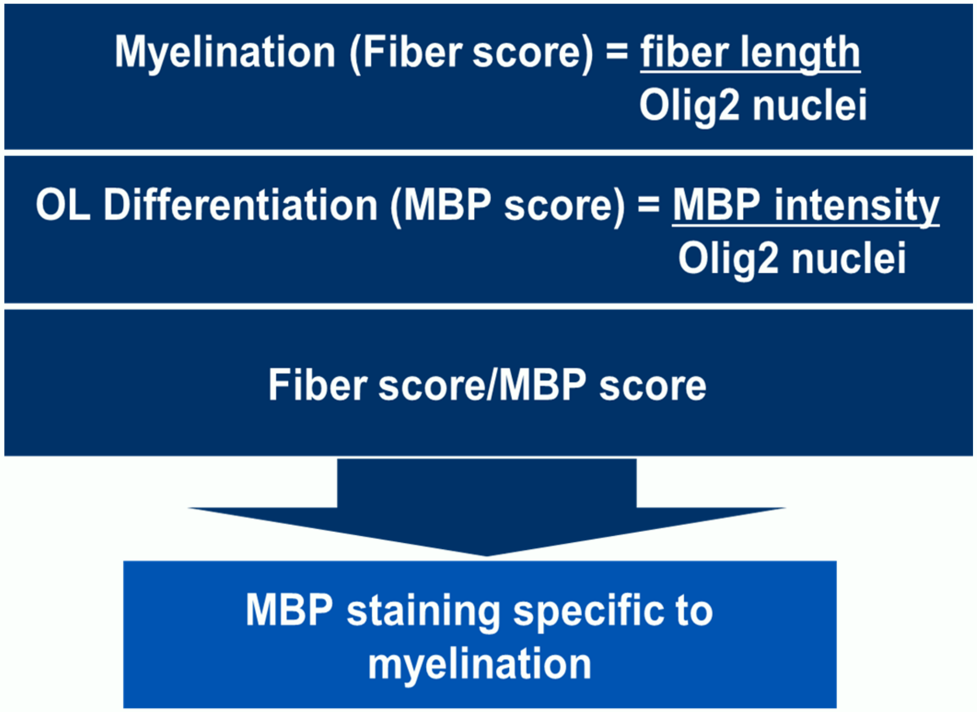

Figure S10. Equations for the quantification of myelination. Schematic figure defining the image quantification calculations derived from MBP intensity mask and number of Olig2 positive cells. OL differentiation is total MBP intensity/Olig2 nuclei and early myelination is calculated as the total length of contiguous MBP staining (fiber length)/Olig2 nuclei. The fiber score/MBP score is a value that normalizes the OL differentiation contribution revealing morphological changes specific to MBP alignment with axons.
