# Supplementary material for: Development of a central nervous system axonal myelination assay for high throughput screening
Source: BMC Neurosci. 2016 Apr 22;17:16. doi: 10.1186/s12868-016-0250-2 (PMC4840960; doi:10.1186/s12868-016-0250-2)
Supplement: Supplementary file 11 — 10.1186/s12868-016-0250-2 Structures, images, and EC50 curves of cortical myelination and OL differentiation hits. [file 12868_2016_250_MOESM11_ESM.pdf]

Fig. S11

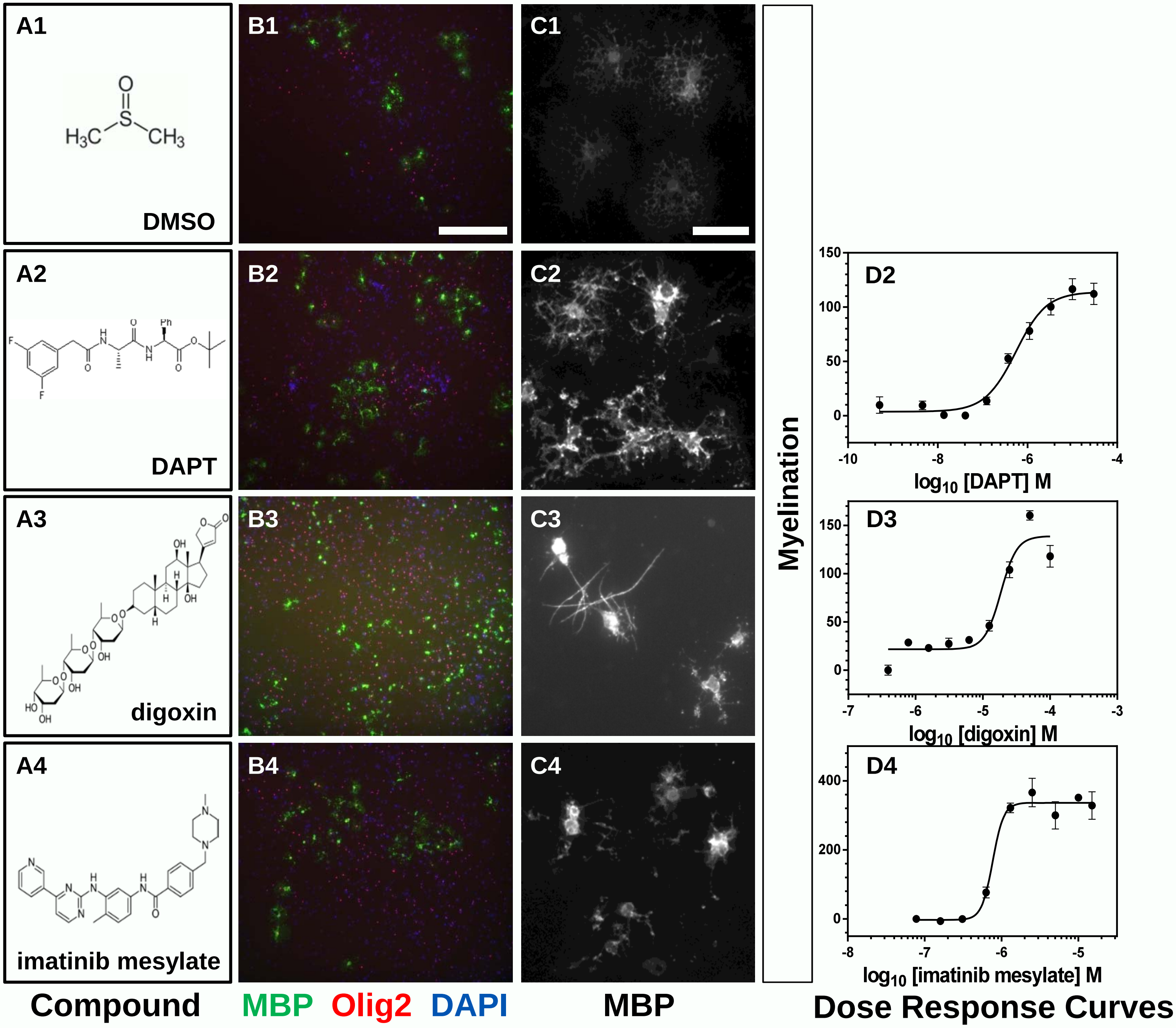
A1
DMSO
A2
DAPT
A3
digoxin
A4
imatinib mesylate
B1
B2
B3
B4
C1
C2
C3
C4
Myelination
D2
log10 [DAPT] M
D3
log10 [digoxin] M
D4
log10 [imatinib mesylate] M
Compound
MBP Olig2 DAPI
MBP
Dose Response Curves

Fig. S11

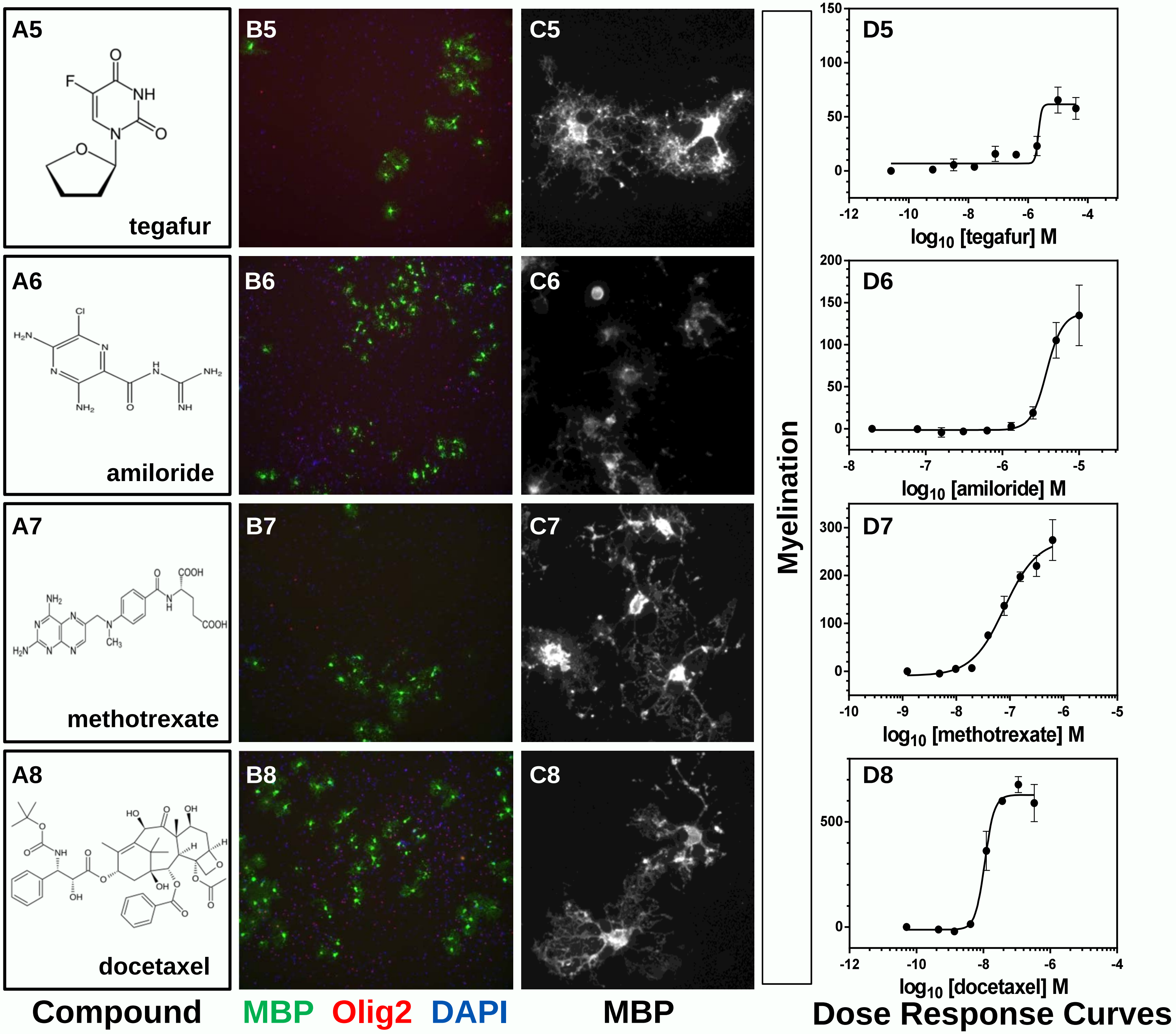

A5
tegafur
B5
C5
D5
log10 [tegafur] M
A6
amiloride
B6
C6
D6
log10 [amiloride] M
A7
methotrexate
B7
C7
D7
log10 [methotrexate] M
A8
docetaxel
B8
C8
D8
log10 [docetaxel] M
Myelination
Compound
MBP Olig2 DAPI
MBP
Dose Response Curves

Fig. S11

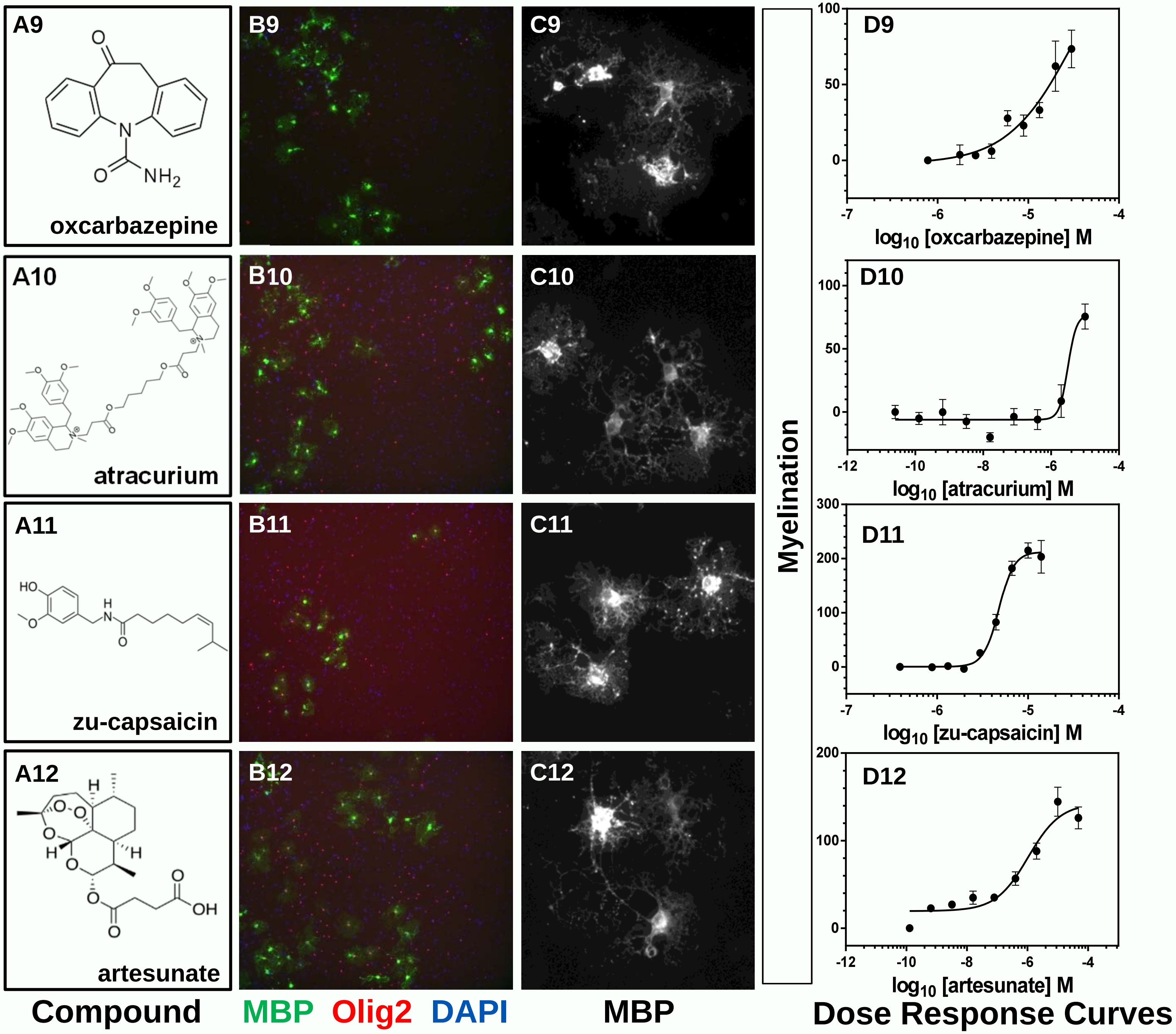

A9
oxcarbazepine
A10
atracurium
A11
zu-capsaicin
A12
artesunate
B9
B10
B11
B12
C9
C10
C11
C12
D9
log10 [oxcarbazepine] M
D10
log10 [atracurium] M
D11
log10 [zu-capsaicin] M
D12
log10 [artesunate] M
Myelination
Compound
MBP Olig2 DAPI
MBP
Dose Response Curves

Figure S11. Structures, images, and EC_{50} curves of cortical myelination and OL differentiation hits. A, Chemical structure and name of each hit compound with the controls, 0.1% DMSO and 1 µM DAPT. B, Example image of each compound directly from the library screening plate at the most efficacious concentration showing MBP (green), Olig2 (red) and DAPI (blue) staining. Olig2 overlapping with DAPI staining appears pink. Bar = 200 µM. C, Enlarged monochrome image (stained for MBP) of each hit from the library screen to highlight OL morphological changes. Bar = 50 µM. D, Representative myelination dose-response curves of each hit (D).
