# Supplementary material for: Development of a central nervous system axonal myelination assay for high throughput screening
Source: BMC Neurosci. 2016 Apr 22;17:16. doi: 10.1186/s12868-016-0250-2 (PMC4840960; doi:10.1186/s12868-016-0250-2)
Supplement: Supplementary file 13 — 10.1186/s12868-016-0250-2 Antibodies. [file 12868_2016_250_MOESM13_ESM.pdf]

Table S2. Antibodies

Antibody	Source	Host	Dilution	Cell Marker
MAP2	Sigma M1406	Mouse	1:500	Neuron/Dendrites
SMI31/32	BioLegend SMI312R	Mouse	1:1000	Neuron/Axons
Neuronal nuclei (NeuN)	Millipore MAB377	Mouse	1:200	Neuron
Iba1	Wako 019-19741	Rabbit	1:1000	Microglia
Glial fibrillary acidic protein (GFAP)	Sigma Ga5	Mouse	1:5000	Astrocyte
Myelin basic protein (MBP)	Abcam ab7349	Rat	1:500	Oligodendrocyte
Olig2	Millipore AB9610	Rabbit	1:1500	OPCs and Oligodendrocyte
O4	Hybridoma supernatant	Mouse	1:50	Oligodendrocyte
GalC	Hybridoma supernatant	Mouse	1:25	Oligodendrocyte
MOG	Hybridoma supernatant	Mouse	1:25	Mature Oligodendrocyte
CNP	Millipore MAB326	Mouse	1:1000	Oligodendrocyte
A2B5	Millipore MAB312	Mouse	1:200	Neural/Glial precursor antigen
Anti-rat Alexa Fluor 488	Molecular Probes	Goat	1:1000	Secondary antibody
Anti-rabbit Alexa Fluor 594	Molecular Probes	Goat	1:1000	Secondary antibody
Anti-mouse Alexa Fluor 594	Molecular Probes	Goat	1:1000	Secondary antibody
